# Diabetes self-management education (DSME) for older persons in Western countries: A scoping review

**DOI:** 10.1371/journal.pone.0288797

**Published:** 2023-08-09

**Authors:** Pilar Camargo-Plazas, Madison Robertson, Beatriz Alvarado, Geneviève C. Paré, Idevania G. Costa, Lenora Duhn

**Affiliations:** 1 School of Nursing, Queen’s University, Kingston, ON, Canada; 2 Department of Public Health Sciences, School of Medicine, Queen’s University, Kingston, ON, Canada; 3 School of Nursing, Lakehead University, Thunder Bay, ON, Canada; National Autonomous University of Mexico, MEXICO

## Abstract

Diabetes mellitus is a chronic metabolic health condition affecting millions globally. Diabetes is a growing concern among aging societies, with its prevalence increasing among those aged 65 and above. Enabling disease self-management via relevant education is part of high-quality care to improve health outcomes and minimize complications for individuals living with diabetes. Successful diabetes self-management education (DSME) programs usually require tailoring for the intended audience; however, there is limited literature about the preferences of older persons in Western countries concerning DSME. As such, a broad overview of DSME for older persons was an identified need. To map the available evidence on DSME for persons aged 65 years and older in Western countries, the JBI methodology for conducting and reporting scoping reviews was used. In this scoping review, we considered all studies about DSME for older persons with T1D and T2D in Western countries where lifestyles, risks, prevention, treatment of diabetes, and approaches to self-management and DSME are similar (e.g., North America, Western and Northern Europe and Australasia). Systematic keyword and subject heading searches were conducted in 10 databases (e.g., MEDLINE, JBI EBP) to identify relevant English language papers published from 2000 to 2022. Titles and abstracts were screened to select eligible papers for full-text reading. Full-text screening was done by four independent reviewers to select studies for the final analysis. The review identified 2,397 studies, of which 1,250 full texts were screened for eligibility. Of the final 44 papers included in the review, only one included participants’ understanding of DSME. The education programs differed in their context, design, delivery mode, theoretical underpinnings, and duration. Type of research designs, outcome measures used to determine the effectiveness of DSME, and knowledge gaps were also detailed. Overall, most interventions were effective and improved clinical and behavioural outcomes. Many of the programs led to improvements in clinical outcomes and participants’ quality of life; however, the content needs to be adapted to older persons according to their culture, different degrees of health literacy, preference of education (e.g., individualized or group), preference of setting, degree of frailty and independence, and comorbidities. Few studies included the voices of older persons in the design, implementation, and evaluation of DSME programs. Such experiential knowledge is vital in developing educational programs to ensure alignment with this population’s preferred learning styles, literacy levels, culture, and needs—such an approach could manifest more substantive, sustained results.

## Introduction

Diabetes mellitus is a chronic metabolic health condition and has been classified as a long-lasting global epidemic [1, 2]. An estimated 536 million people currently live with diabetes, a number that is expected to increase to 643 million by the year 2030 and 783 million by the year 2045 [1]. Every 5 seconds, someone dies as a result of diabetes and its complications [1]. Type 1 diabetes (T1D) is caused by the autoimmune destruction of β-cells in pancreatic islets as a result of genetic and environmental factors [3]. Type 2 diabetes (T2D) occurs primarily when there is defective insulin secretion by pancreatic β-cells, and normally insulin-sensitive tissues are unable to respond to insulin [4]. Across the globe, ageing societies are facing the challenges of diabetes mellitus and its complications [2], as its prevalence is growing among those 65 years of age and older [5, 6]. In 2019, over 136 million older adults (65–99 years old) were living with diabetes—a number that is expected to increase to 195 million by 2030 and 276 million by 2045 [5]. Older persons with diabetes have a high prevalence of diabetes-related complications and diabetes-associated conditions resulting in multimorbidity, frailty and disability [5, 6]. Diabetes is a public health burden as it increases expenditures for national and social systems [5]; in 2021, the global health expenditure of diabetes was estimated at $ 966 billion USD [1]. Advancements in diabetes care have also increased the life expectancy of older persons with T1D [7, 8]. T1D is a growing but still under-explored concern in older adults [7, 8]. Due to economic development, rapid changes in lifestyles, and an increasingly ageing population, T2D is the most common type of diabetes mellitus in older populations [6, 9, 10].

Despite the growing problem of diabetes, researchers have shown its management in older persons is particularly complicated and far from optimal [11–14]. In older populations, inequities are compounded by additional differences in risk factors, such as multimorbidity, polypharmacy, cognitive decline, disability, frailty, and socioeconomic factors [15–20]. An increasing proportion of older persons with diabetes are living alone in communities, dealing with their condition with minimal or no support [21]. Furthermore, diabetes care involves making daily self-management decisions and performing complex self-care tasks, which require visual, motor, cognitive, and executive skills and problem-solving and coping strategies [22, 23]. Therefore, ongoing diabetes education, support for self-management, and regular monitoring are crucial to reduce the personal and social impacts of diabetes among older persons [9, 10, 24, 25].

Diabetes self-management education (DSME) is critical in the ongoing treatment of people with diabetes and those at risk of developing the disease [9, 11, 14, 17, 20]. DSME involves a variety of behavioural, psychosocial, and psychological interventions and a combination of empowering, didactic, interactive, and collaborative educational methods [9, 17, 18, 26–29]. The content of DSME can be structured [30, 31], person-centred [13, 14, 29], or empowering [9, 10, 17]. There are varied and well-established DSME programs [30–32].

Researchers have demonstrated the value of DSME in improving learning [9, 15, 33], behavioural outcomes [34, 35], and clinical outcomes [35–38]. Systematic reviews have been conducted about the effectiveness of self-management education for: glycemic control [39, 40]; community-dwelling older adults [41]; the use of technology and its effects on diabetes outcomes [42]; people from diverse backgrounds [43]; women [44]; and individuals in low-income settings [45]. However, little is known about the implementation of DSME in adults aged 65 years and older or the range of DSME programs/interventions for this population [46]. Furthermore, in previous DSME studies, people aged 65 years and over are often underrepresented; this is a problematic knowledge gap for the development of evidence-informed guidelines targeted toward this unique population [22, 23]. With the increasing evidence about the importance and variety of diabetes self-management interventions, a comprehensive overview of DSME for adults aged 65 years and older with diabetes is warranted to identify the knowledge and methodological deficits in this area of study [46]. Therefore, the objective of this scoping review was to map the available evidence on DSME for persons aged 65 years and older in Western countries.

## Methods

This scoping review was conducted following the JBI methodology framework for scoping reviews [47], and we adhered to the Preferred Reporting Items for Systematic Reviews and Meta-Analyses Extension for Scoping Reviews (PRISMA-ScR) checklist [47] (S1 Appendix). Our published protocol [46] was registered through Open Science Framework and is available at https://doi.org/10.17605/OSF.IO/W4KCQ. Our overall review question was: What has been reported in the literature about DSME for older persons aged 65 years and older living with T1D and T2D? To address this, seven sub-questions were also detailed. Following the PCC (Population (or participants)/Concept/Context) framework as recommended in the JBI methodology, our population was older persons with T1D and T2D, the concept was DSME for older persons with diabetes, and the context was all Western countries.

### Inclusion and exclusion criteria

In this review, we considered studies involving older persons with T1D and T2D. As this work focused on education after a diagnosis of diabetes, studies with participants with prediabetes were excluded. Older persons were defined as those 65 years of age and older. Globally, the number of older persons aged 65 years and over is projected to increase [1]. People aged 65 years and over have been frequently excluded from research (e.g., clinical trials); when included, researchers often do not report analysis by age [48, 49]. Our review was not specific to gender, sex, ethnicity, or frailty [46]. Studies including older persons with and without diabetes were considered when separate analyses for people with and without diabetes were provided [46]. Studies mixing older and younger participants were considered when separate analyses for the older and younger groups were provided [46].

In this scoping review we considered all studies about DSME for older persons with T1D and T2D in Western countries (e.g., North America, Western and Northern Europe and Australasia) where lifestyles, risks, prevention, treatment of diabetes, and approaches to self-management and DSME are similar. Countries included in the review were Australia, Austria, Belgium, Canada, Denmark, France, Finland, Germany, New Zealand, Norway, Sweden, Switzerland, the Republic of Ireland, The Netherlands, the United Kingdom and the United States. DSME refers to the tailored individual skills and information people learn and apply to manage their diabetes. DSME is guided by evidence-based standards [50]. The goal of DSME is to engage and empower people with diabetes to navigate self-management decisions and activities to control their condition and to avoid complications [51]. We considered studies that provided information about DSME for older persons. Approaches to DSME include structured or formal diabetes education, online or electronic delivery programs, behavioural/psychological approaches, theory-based programs, empowering strategies, problem-solving emphasis, and approaches focusing on psychosocial strategies [46].

We considered a wide range of quantitative, qualitative, and mixed-method designs. Experimental and quasi-experimental study designs were included (e.g., randomized-controlled trials [RCTs], and nonrandomized pre-and post-test designs), in addition to analytical observational studies, including prospective and retrospective cohort studies, population-based studies, and analytical cross-sectional studies. Furthermore, we considered descriptive observational study designs, including clinical case and descriptive cross-sectional studies. Studies and projects about quality improvement and professional-led interventions were included. Qualitative studies included, but were not limited to, traditions, such as phenomenology, grounded theory, ethnography, critical theory, feminist, descriptive and exploratory designs, case studies, and participatory action research. In addition, eligible systematic reviews were considered. Program descriptions, clinical reviews, text (e.g., textbooks), guidelines, practice briefs, and opinion papers were also included.

We included studies published in English. The period considered was from the year 2000 to the dates of the searches (August 2022 for bibliographic database searches and February 2023 for the final searches of new sources). We decided not to include articles published before 2000 because of the rapid evolution of DSME in the past two decades [46].

### Search strategy

We identified published primary studies, text and opinion papers, and grey literature dedicated to the topic of DSME in older persons. We conducted searches electronically and manually; the latter was conducted by searching for relevant articles or papers in the reference lists of the selected articles. A three-step search strategy was used in this review. An initial limited search of MEDLINE was performed to identify articles about the topic, followed by an analysis of the text contained in the titles and abstracts of the retrieved papers and of the keywords used to describe the articles. The research team discussed and developed the most appropriate keywords and synonyms for search activities using feedback from an academic librarian. Keywords for the search included: diabetes mellitus, diabetes, diabetes type 1, diabetes type 2, diabetes education, diabetes training, diabetes knowledge, health education, health literacy, health promotion, diabetes training, older adults, elderly, geriatric, geriatrics, aging, senior, seniors, older people, aged 65 or 65+, self-management, self-care, self-regulation, and self-monitoring (see S2 Appendix). Boolean operators (OR, AND), including adjacencies and truncations, were used to combine appropriate keywords and related terms. This was followed by a second search across all included relevant databases using all keywords and index terms, and the third and final step of searching for additional studies by appraising and screening the reference list of identified reports and articles. The search was conducted in June 2020 and was updated in February 2023. The following databases and organizations for health care disciplines were searched: MEDLINE, PubMed, Google Scholar, ProQuest Dissertations and Theses, World Health Organization, PsycINFO, JBI EBP, Cochrane Databases of Systematics Reviews (all via OVID), CINAHL (EBSCO), and Pre-CINAHL (EBSCO). In addition, we searched conference proceedings of international conferences on diabetes, geriatrics, and gerontology associations and society meetings (see S2 Appendix).

### Selection criteria

#### Study selection and data extraction

Four independent reviewers extracted data from the included records using a data extraction tool developed by the research team [46]. The data extraction tool was not modified or revised during the extraction process. Data extraction was derived from our overarching research question and sub-questions [46]. Any disagreements between the reviewers were resolved through discussion. The following data were collected:

- Study characteristics (e.g., country, publication date, study design and purpose, sample size).
- Participant characteristics (e.g., mean age, diabetes duration, gender).
- DSME characteristics (e.g., type of program, number of sessions, setting, facilitator, theoretical/philosophical underpinning of the program, topics covered, program length).
- DSME outcomes measures, results and gaps identified by authors.

#### Data analysis and reporting

We applied descriptive analysis of the extent, nature, and distribution of the studies included in the review and a narrative summary of the data collected. This was accomplished by summarizing the literature according to the characteristics of the studies, the characteristics of the DSME programs/interventions and outcomes, and the gaps identified by the researchers. We mapped the extent, range, and nature of the research about this topic using visual representations of the data. Data were charted, categorized, and summarized, and we explored similarities and differences within and between studies to identify the robustness of the included records. Herein, we reported quantitative (e.g., frequency) and qualitative results in a manner that aligned with the objective of this scoping review.

## Results

### Study inclusion

**Fig 1:**
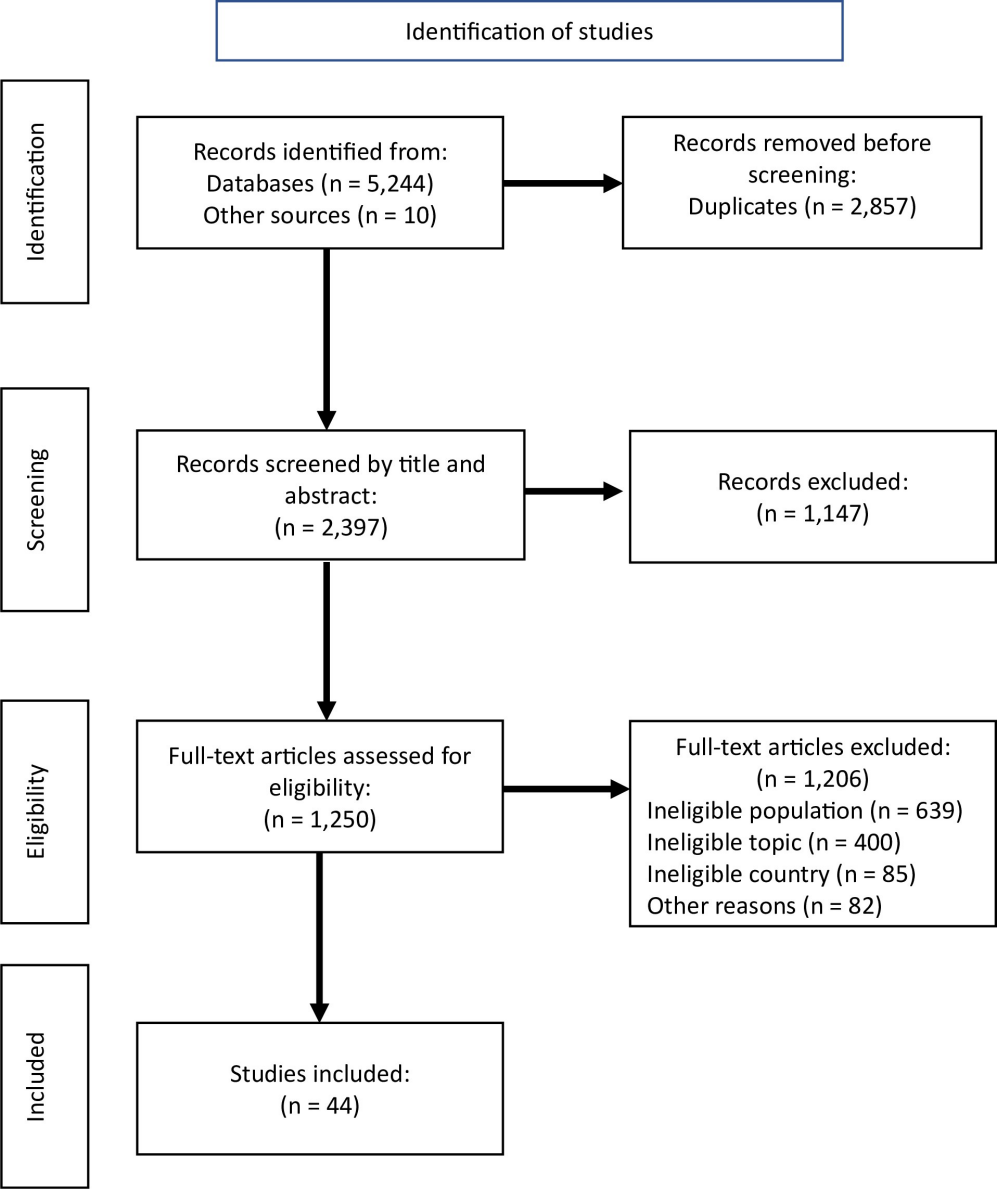
Stages of study identification and selection.

From the database searches we retrieved 5,254 records. After duplicates were removed, the remaining 2,397 records were screened by title and abstract, and 1,147 were excluded. The full texts of 1,250 articles were assessed for eligibility, and another 1,206 articles were excluded (reasons are documented in Fig 1). The most common reasons for paper exclusion were ineligible population (n = 639) and ineligible topic (n = 400). A final total of 44 records were included in this review. Fig 1 is a depiction of the stages of study identification and selection.

### Characteristics of included studies

Details of the 44 studies are summarized in Tables 1–3. All articles were published in the past 22 years (2001 to 2022). Most of the studies were conducted in the United States (n = 19), followed by Canada (n = 11), Australia (n = 3), the United Kingdom (n = 3), Germany (n = 3), and the Netherlands (n = 2). The remaining studies were conducted in Belgium (n = 1), Sweden (n = 1), and Norway (n = 1). Thirty-nine studies (91%) were about T2D in older persons, one study included older persons with T1D [52], and five studies did not specify the type of diabetes [11, 17, 20, 36, 53]. The total sample size for the 44 studies was 63,230 participants with a mean age of 69.06 years and a mean duration of diabetes of 9.85 years (excluding two studies without sample characteristics and six studies without information about the mean age of the study participants).

**Table 1:** Details of the reviewed papers.

Author	Country	Purpose and Study Design	Sample Description	Conclusions and Recommendations
Akhter et al. [9]	United Kingdom	To assess the acceptability of empowerment-based DE for patients with T2D. Prospective cohort with non-equivalent control study of an empowerment approach to T2D education, prior to RCT.	578 participants. Diabetes duration 9±11 years.* Age 65±10 years. Males = 56.7%. Attended diabetes education sessions before = 54.8%. Non-British origin = 7.6%.	DSME using an empowerment approach increases knowledge; however, it was difficult to evaluate the impact of empower education on metabolic control. Reinforce DSME after diagnosis. Demand for knowledge about food/diet highlights a gap in existing services.
Andrich et al. [10]	United States	To improve glycemic control and quality of life (QoL) of older adults with T2DM. Quality improvement project. One group pretest-posttest design.	24 participants, 9 female (37.5%) and 15 male (62.5%). Mean age was 74 years. Mean DM duration = 7.5 years.	DMSE improves glycemic control and QoL. Improve the education of health care providers (HCPs).
Babalola et al. [15]	United States	To increase diabetes knowledge, improve self-management behaviours (DSM) and glycemic control among older aged Mexican Americans. Quality improvement. One group, pre-test-post-test design.	12 participants. Mean age = 68.3 years. 83% were females, and 17% were males. Mexican Americans.	Culturally-competent DSME can positively impact knowledge, DSM behaviours and glycemic values for participants.
Bastiaens et al. [60]	Belgium	To develop and implement a group DSME program for people with T2DM in primary care. One group pretest-posttest design.	44 participants. 53% were male. Mean age = 66 years, and mean DM duration = 5 years.	A group education program in primary care is feasible and effective. Need to study the education process in greater depth.
Bowman & Epp [65]	Canada	To evaluate outcomes of diabetes care in two communities in rural Manitoba, Canada. Static group comparison. Cross-sectional survey.	78 respondents. Mean age of 68.5 years. 89.7% were Caucasian.	Rural DE has a positive effect on knowledge, DSM and self-efficacy. A shift from a biomedical paradigm toward a health promotion approach is needed.
Braun et al. [16]	Germany	To evaluate structured treatment and teaching program (TTP) for geriatric patients with impaired cognitive function. Randomized controlled trial (RCT).	102 participants. Mean age = 68.6 years. Mean DM duration = 10.3 years. Female = 54.9%.	Structured treatment and teaching programs (TTP) improved metabolic control, and DSM in older participants with cognitive disabilities. Mental health in older persons with DM should be screened throughout. HbAC1 must be controlled before participation in education program.
Braun et al. [61]	Germany	To evaluate the impact of insulin therapy, metabolic control and structured patient education on the QoL in insulin-treated patients with T2DM Pre-test post-test with control group.	71 participants. Mean age = 68.9 years. Mean DM duration = 11.2 years.	DTTP program resulted in better metabolic control and improved QoL in older participants.
Braun et al. [38]	Germany	To evaluate the effectiveness of a new structured diabetes teaching and treatment program (DTTP) with specific didactical approaches and topics for geriatric patients with DM. Prospective, RCT (multi-centre).	155 patients. Mean age = 76.2 years. Female = 66.5%. Mean DM duration = 12.5 years.	The new DTTP is effective in improving metabolic control and maintaining DSM. Evaluate a culturally tailored education to address the causal effects of the intervention in other ethnic groups.
Camargo-Plazas et al. [17]	Canada	To recommend the integration of Paulo Freire’s culture circles in the development of DSME programs for seniors. Practice brief.	Not applicable.	Freire’s method has the potential to generate more effective DSME programs for older adults.
Choi et al. [59]	United States	To assess the effectiveness, feasibility and acceptability of a culturally-tailored community-based DSM program for Korean immigrants. One group pretest-posttest design.	41 participants. Mean age = 70 years. Mean DM duration = 8.9 years. Male = 46.3%.	Culturally-tailored DSME is an effective, feasible and accepted approach to improving diabetes outcomes in participants with limited access to mainstream clinic-based DSM programs.
Fritschi et al. [18]	United States	To explore self-regulation skills with real-time activity and glucose monitoring among Black women with T2DM. One-shot case study. Mixed study. Small acceptability trial.	8 participants. Mean age = 68 years. Mean duration DM = 15.3 years.	The use of personalized education, continuous glucose monitoring and Fitbit activity tracker was associated with self-regulation behaviours.
Gorter et al. [24]	Netherlands	To assess the preferences of patients with T2DM regarding self-care activities and DE. Cross-sectional survey, static group comparison.	994 participants. Mean age = 65 years. Male = 54%. 56.4% were living with diabetes for more than 5 years. 97% Western ethnicity.	Education should be individualized and part of regular diabetes check-ups. HCPs should be aware of specific barriers to lifestyle advice. Regarding group or individual education, HCPs may consider asking patients about their preferences to make a tailored education plan. HCPs should be aware of specific barriers to lifestyle advice.
Hunt et al. [33]	United States	To develop, implement and evaluate the effectiveness of DSME modules delivered via iPad devices to increase self-management knowledge (SMK) evels in adults living with T2DM in rural areas. One group pre-test-post-test design. Pilot test intervention.	30 participants 19 female, 11 male, Mean age was 73 years. Mean duration of DM 11 years.	Diabetes knowledge increased significantly after the education intervention. Lower SM dietary scores indicated difficulty maintaining healthy eating habits. Investigate the type and quality of education received from HCPs.
Kellow et al. [34]	Australia	To inform the development of a patient-centered educational decision aid for diabetes care: *SEE-Diabetes (Support-Engage-Empower-Diabetes)*. One group pre-test-posttest design.	34 individuals. Male = 35%. Five education sessions. Mean age = 69.1 years. Mean duration of DM = 10 years.	A culturally-tailored program reduced waist-to-height ratio and waist circumference and increased diabetes self-management behaviour (DSMB) while reducing diabetes distress.
Kemper et al. [19]	United States	To determine if persons with less than a high school education had less knowledge related to diabetes compared to persons with at least a high school education. Cross-sectional descriptive study.	28 participants. Mean age = 70.5 years.	DSME must consider the health literacy of the target population. Participants without a high school diploma received less formal education than those with a diploma. Assess learning needs and provide appropriate educational material to meet the learning needs and maximize comprehension.
Kjellsdotter et al. [61]	Sweden	To describe patients’ experiences of group-based education using the *Taking charge one’s life with type 2 diabetes* model. Qualitative approach with a phenomenological perspective.	12 participants (7 men and 5 women). Mean age = 71 years.	Group-based education is feasible. Participants appreciated form of education that supports and facilitates learning through reflection and dialogue.
Knott et al. [32]	United Kingdom	To describe how a Primary Care Trust (PCT) program was transferred to three community settings without affecting clinical outcomes (e.g., HgAC1, BMI). Nurse-led, open access, structured education program.	540 hospital patients (230 in PCT1 [local community hospital] and 228 in PCT2 [local community centre]). Mean age = 66 years in all three settings.	Structure education is an important aspect of diabetes care. A well-established program has successfully transferred to the community without affecting clinical outcomes.
Knox et al. [35]	United States	To evaluate the impact of a peer support program on the health outcomes of patients already receiving well-organized, comprehensive diabetes care. Mixed-method, nonrandomized pre-post with control-group.	485 participants. 82% were 65 years or older. Female = 62%. 41% = Anglo; 45% = Hispanic; 7% = Black.	The addition of peer mentoring to an already well-organized comprehensive diabetes care program does not improve outcomes. Evaluate the impact of emotional distress and the effects of a community-based peer mentoring program on family members.
Liu et al. [37]	United States	To evaluate the influence of DE program in a primary care setting. One group pretest-posttest design.	220 adults. Mean age = 65.7 years. 60% = female. 60.5% = African American.	Completion of 3 or 4 sessions of diabetes education (DE) resulted in improvements in metabolic parameters, lipid profile, BP and weight loss and increased use of hypertension and cholesterol medications. Include people with different ethnicities and cultural backgrounds, noncompliant patients, patients who were no referred to the DE program, and patients who successfully finished programs.
Liu et al. [52]	Canada	To examine patient and paramedic experiences with a direct electronic referral program for hypoglycemia education postparamedic assist-requiring hypoglycemia, including barriers to program referral and education attendance. Mixed-method (Qualitative and Quantitative) (cross-sectional survey).	14 older adults participated in the education session and consented to interview. Mean age = 66 years. Mean duration of DM = 30 years. Male = 57%. 57% were T1DM.	Program uptake was low as patients felt DE was helpful in the early stages of their disease but of fewer benefits at later stages. DE was unnecessary or repetitive. Evaluate patient-centred strategies that may help improve focused hypoglycemia education attendance post severe hypoglycemia episode. Address diabetes stigma and negative emotions surrounding diabetes education.
Luo et al. [58]	United States	To assess rural-urban differences in participation rates of DSME and associated factors among Medicare beneficiaries with T2DM. Static group comparison. Cross-sectional design.	3,799 beneficiaries aged 65 years and older with self-reported T2DM.	The findings highlight the challenges to achieving widespread dissemination and uptake of DSME. Continuous efforts are needed to assist rural residents in engaging in DSME training to reduce the burden of DM. Evaluate the current contents and effectiveness of different delivery modes of DSME.
Markle Reid et al [14]	Canada	To compare the effect of a 6-month community-based intervention with that of usual care on QoL, depressive symptoms, anxiety, self-efficacy, self-management, and health care costs in older adults with T2DM and 2 or more comorbidities. Multisite, single-blind, parallel, pragmatic, RCT.	Community-dwelling older adults (≥65 years) with T2DM and 2 or more comorbidities randomized into intervention (n = 80) and control (n = 79) groups.	Participation in a 6-month community-based intervention in Ontario improved QoL and reduced depressive symptoms without increasing total health care costs. Intervention sustainability should be considered given many studies show only short-term effects.
Marsh et al. [36]	United States	To develop an innovative care delivery model for rural health care organization for vulnerable and marginalized populations with chronic illnesses. One group pre-post intervention design, quality improvement project.	12 participants. Mean age = 68 years. Female = 75%. White = 66.7%; Black = 33.3%.	Community health worker (CHW) home visits and clinical team telemedicine appointments reduce the burden diabetes management creates for underserved patients aged ≥65 years, while improving learning, behavioral and clinical outcomes. This intervention reduced the impact of the SDH by immersing the CHW in the community and clinical setting. The patients and HCPs were highly satisfied with the project interventions. Examine the cost-effectiveness, long-term feasibility and overall impact of DSME-guided CHW and telemedicine interventions in other health settings.
Miklavcic et al. [13]	Canada	To evaluate the effect of a community-based intervention versus usual care on physical functioning, mental health, depressive symptoms, anxiety, self-efficacy, self-management, and health care costs in older adults with T2DM and 2 or more comorbidities. Multisite, single-blind, parallel, pragmatic, RCT	132 participants (70 = Intervention; 62 = Control). 42% = 75+ years. Female = 55%. Over 75% had at least 6 chronic conditions.	The pragmatic trial of an interprofessional, intersectoral self-management (SM) intervention for older adults in Alberta demonstrated inconclusive results for physical and mental functioning.
Millar et al. [66]	Canada	To determine whether attending DE is associated with blood glucose self-monitoring among unselected older adults in routine clinical care. Static group comparison. Cross-sectional study.	15,190 people (attending group (GA) = 2024 and non-attending group (GNA) = 13,166). Mean age = 74.01 years (GA) and 68.9 years (GNA).	DE is associated with self-management behaviour (SMB)in real-world clinical care. These findings support the effectiveness of DMSE programs in increasing self-care behaviours
Miller et al. [63]	United States	To evaluate the impact of a nutrition intervention on the blood glucose and lipoprotein levels of adults 65 years and over without functional limitations but with T2. RCT.	92 people. 53% = Female; 47% = Male. Mean age = 73 years for control group and 72.1 years for experimental group. 83% = Caucasian. 66% had high school diploma.	Nutritional education can improve metabolic control among the cohort of participants. Improved metabolic outcomes reduce the morbidity and mortality associated with DM. Education must be of sufficient duration and quality to facilitate self-management practices.
Miller et al. [31]	Australia	To evaluate the effects of Diabetes Education and Self-Management for Ongoing and Newly Diagnosed (DESMOND) program on patient activation in adults living with T2DM. Intervention study. One group, pre-test-post-test design.	233 participants. Mean age = 66 years. Male = 35.6%; 63.1% = Female. 67% were living with diabetes 12 months and more.	DESMOND program increased patient activation in a real-world setting.
Murray et al. [53]	Canada	To evaluate the effect of DE program attendance, which provides patients with DSME, on prescriptions for cardiovascular risk reduction, diabetes treatment, and visits for retinopathy screening. Population-based cohort study.	22,606 attendees. Mean age = 73.8 years. Female = 50.4%. 76.4% were living with diabetes more than 5 years.	DSME is associated with better Quality of Care (QoC) in the elderly in Ontario.
Narindrarangkura et al. [55]	United States	To identify the information needs of people with diabetes aged 65 and older through surveys and focus group to inform the development of a patient-centered educational decision aid for diabetes care: *SEE-Diabetes (Support-Engage-Empowerment-Diabetes)*. Mixed-methods design. (Qualitative ([focus groups)] and Quantitative [(cross-sectional survey]) methods.	37 participants responded to the survey (response rate 45%). Mean age 66 years (24–82 years, SD = 12 years). Female = 22 (60%). Mean DM duration = 20.9 years. White = 95%; Asian = 2.7%; African American = 2.7%.	Online accessible clinic notes were helpful for patients. The readability of notes used for DSME must be improved.
Pauley et al. [25]	Canada	To supplement traditional diabetes education typically provided by a patient’s in-home nurse to one wherein coaching support is delivered by a personal support worker (PSW). RCT.	94 participants. Male = 46. Mean age = 66.9 years (control group) and 65.1 years (experimental group). Years since diagnosis = 20.3 years (controls) and 15.6 years (experimental group).	PSWs may not confer additional benefits for improving self-efficacy in the context of a DSM intervention. A PSW coaching intervention may be sufficient to improve depression among those demonstrating an increased depression burden. Further study of PSWs as an extension of a collaborative approach to DSM as a depression mitigation intervention is needed.
Redmond et al. [56]	United States	To improve participant AC1 levels (particularly in those with high initial AC1 concentrations), diabetes self-management activities, and AC1 knowledge. One group pre-test-post-test design.	Sample size = 91. Mean age = 73 years. Caucasian = 60%, Black = 40%. Living with diabetes 0–10 years 66 and more than 11 years 34.	This intervention improved DSM and AC1 knowledge, with concurrent decreases in AC1 levels. More dietary interventions for older adults with diabetes are needed.
Robertson et al. [20]	Canada	To explore DSME experiences of older adults with diabetes in rural Ontario. Participatory action research, art-based and hermeneutic phenomenological study.	14 participants. Mean age = 74 years. Male = 11; Female = 3. Mean time since diabetes diagnosis = 15.7 years. 85% = English ethnicity.	Although some participants recognized the importance of DSME and its availability in their community, they felt they did not need it, believing diabetes does not have a large effect on their everyday lives. Living in rural setting was a barrier to access DSME. Sources of information for DE included: 1) independent research (e.g., reading online, books, booklets); 2) learning from family and friends with diabetes; and 3) asking HCPs. DSME should account for older adults’ preferences in learning about diabetes and self-management to promote access to evidence-based information, bolster knowledge and self-management efficacy, and improve disease control.
Rygg et al [64]	Norway	RCT. To evaluate the efficacy of ongoing group based DSME for patients with T2D.	146 patients. Mean age = 66 years. Male = 55%. Median time since DM diagnosis = 5 years.	The locally developed DSME programs prevented an increase in AC1 in patients with higher AC1 levels. The intervention group showed better diabetes knowledge and improved DSM skills after the program. HCPs should critically evaluate locally developed programs and compare their content to programs developed for studies and in guidelines.
Sanchez [26]	United States	To implement DSME in primary care using the Chronic Care Model and shared medical appointments (SMA) to provide evidence-based interventions to improve process and measure outcomes. One group pretest-posttest design. Quality improvement project.	70 Mexican American participants. Mean age = 66 years.	A SMA program can increase revenue and productivity. Also, it improved DSM and increased provider and patient satisfaction.
Shah et al. [11]	Canada	To assess its effectiveness to improve quality of care and reduce the risk of long-term diabetes complications in unselected older patients with recently diagnosed diabetes in routine clinical care. Population-based cohort study.	Population-level health care administrative databases and registries (n = 16,520 individuals). Mean age = 73.1 years. Males = 7,852 males; Females = 8,668.	In real-world clinical care, DSME for older patients with recently diagnosed DM was associated with modest improvements in quality of care (QoC), but not reductions in long-term clinical events. Need for evaluation of DSME programs to ensure they are improving patient outcomes.
Simmons et al. [27]	United Kingdom	To test the feasibility and acceptability of the planned individual and group support programs, as well as to test the procedure to recruit trial participants (as peer supporters and as peers in the program). RCT pilot study.	6 participants. Mean age = 67.6 years. Mean time of living with DM = 9.1 years.	Recruitment to a full trial of peer support within the existing study design is feasible with some amendments. Attendance emerged as a key issue needing close monitoring and additional intervention during trial.
Smith et al. [54]	Australia	To document clients’ perspectives on the value of their participation in group education and to explore these perspectives in participants’ own words, giving clients a voice, to demonstrate that group education sessions are seen as valuable for those living with T2DM. Single case study (Qualitative).	15 participants. Female = 11; Male = 4. White = 14; Other = 1.	This study proved clients valued group education sessions for the social contact, the increase in knowledge about the disease, the help with self-management, and the support—factors recognized as important to maintaining health. Group education is a cost-effective method for DSM that funders need to consider.
Suhl & Bonsignore [22]	United States	To review the principles that emerge from the AGS and AADE guidelines and present practical application for clinical practice. Clinical case study.	One participant. Aged = 78 years. Ethnicity = Russian.	DSME for older adults is complicated by the high prevalence of medical comorbidities and declining functional status among this patient population. DSME should be individualized and multidisciplinary, involving caregivers and patients, and should carefully weigh the potential effects of diabetes treatments on QoL.
Sullivan et al. [28]	United States	To provide health coaching to patients with a primary or secondary diagnosis of T2DM to increase self-management (SM) skills and reduce 30-day readmissions. One group pretest-posttest design. Program development.	20 participants. Mean age = 67 years.	Health coaching that emphasized SM empowered patients to set healthy goals and provided support through weekly reminders to improve SM for patients with T2DM.
Tessier & Lassmann-Vague [23]	Canada	To explore the impact of education through a multidisciplinary approach on glycemic control in a specific population of older individuals with DM. Literature review.	Not reported.	DSME should be viewed as a long-term process where goals should be discussed with older adults and caregivers. Education should be individualized based on the diversity of older populations. Interventions should be directed toward the population with the best life expectancy, but without neglecting the most vulnerable ones.
Van Vugt et al. [30]	Netherlands	To test whether low well-being modifies the effects of the PRISMA self-management education (SME) program (Dutch DESMOND). One group pretest-posttest design.	297 participants. Patient were grouped in low (n = 63) and normal well-being (n = 234). Mean age = 67.3 years. Female = 144; Male = 153. Mean DM duration = 8.42 years. Dutch Caucasian = 96.6%.	The PRISMA SME (Dutch DESMOND) seems as effective for people with normal well-being as for people with low well-being. PRISMA programs with ethnic minorities warrant future research.
Walter et al. [29]	United States	To assess the effectiveness of a single Conversation Map session led by a pharmacist through a pre-test/post-test study design. One group pretest-posttest study design.	32 veterans. Mean age = 65 years. Mean DM duration = 5 years.	This study showed favourable results regarding the use of the Conversation Map tool for small-group diabetes education. Improved attitude about diabetes management. Use the Conversation Map on newly diagnosed people with diabetes and address long-term retention of knowledge and assessment of patient-oriented outcomes.
Whitehouse et al. [57]	United States	To compare outcomes for older adults with T2DM and obesity following participation in a transitional care intervention that included DSME and homecare. Static group comparison. Secondary analysis.	180 participants.? Mean age for those who had inpatient DSME = 65.85 years; Mean age for those with inpatient DSME + homecare = 69.89 years; Mean age of controls = 68.65 years. African American = 82%; White = 15%.	Results support a transitional care education intervention for older adults with T2DM and obesity. DSME is beneficial. The content of DSME needs to be adapted to older adults with different degrees of independence and comorbidity.
Whitehouse et al. [12]	United States	To investigate the feasibility of telehealth-delivered DSME and support for older adults with T2DM. One group pretest-posttest design study (Pilot).	20 participants (12 completed the intervention). Mean age = 66.5 years. Mean DM duration = 13.8 years. Black = 75%; White = 20%; Asian = 5%.	DSME delivered via telehealth is a feasible and acceptable method. The cost of telehealth education should be investigated.

* Diabetes duration in mean years = 9 (SD = +/-11)

**Table 2:** Details of the DSME programs.

Author, Year	Type of Program, Number of Sessions	Setting	Facilitator	Theoretical/Philosophical Underpinning	Topics	Program Length
Akhter et al. [9]	Empowerment education. One single education session—3.5 hrs followed by question/answer (QA) session.	Community	Diabetes Educator (DE), Registered Dietitian (RD), nurses.	Empowerment underpinnings.	Identifying carbohydrates and understanding portions, truths and myths about diabetes, knowing your numbers and medications, keeping active and looking after your feet.	Not reported.
Andrich et al [10]	DSME support and goal-setting sessions. 1 education session—20 minutes during a 3-day period.	Clinic	Advance Registered Nurse Practitioner (ARNP).	Plan-Do-Study-Act (PDSA) cycle.	Information sharing about disease management, psychosocial support as it relates to disease management, behavioural support in managing T2DM, including glucose monitoring, diet, and lifestyle modification, multidisciplinary integration of care, and care coordination including referrals to an optometrist, RD, or podiatrist as needed.	3 months.
Babalola et al. [15]	Culturally sensitive, age-specific intervention. Single series of 4 sessions every other week.	Clinic	Principal investigator.	Health Promotion Model. Cultural competency criteria.	Identification of critical barriers to diabetes self-care behaviours. DE content was based on the American Association of Diabetes Educators (AADE) and the American Diabetes Association (ADA) standards of care.	3 months.
Bastiaens et al [60]	Group program. Five 2hr sessions and 1 follow-up meeting after 3 months.	Primary care	DE, nurses, psychologist.	Chronic Care Model.	Healthy eating and physical activity.	3 months.
Braun et al. [16]	In-patient structured Treatment and Teaching Programs (TTP). 5 days, 20 education hours.	Clinic	DE, nurses.	Not reported.	Conventional insulin therapy—individual treatment goals and patient motivation versus nutrition, insulin injection, urine/blood glucose self-monitoring acute and late complications.	6 months.
Braun et al. [62]	Structured patient education. 5 days, 15 education hours.	Clinic	Not reported.	Not reported.	Instruction in insulin injection, self-monitoring, hypoglycemia, acute/late complications, nutrition, and foot care.	6 months.
Braun et al. [38]	Structured patient education. 7 classes of 45 minutes each.	Clinic	Not reported.	Not reported.	Intensive training of practical capabilities, such as insulin injection, self-monitoring and management of hypoglycemia.	6 months.
Camargo-Plazas et al. [17]	Empowerment education and culture circles.	Community	Not reported.	Paulo Freire’s method of education.	Client-centred education and flexible.	Not reported.
Choi et al. [59]	Culturally-tailored education. 2 sessions (first session lasted 1.5hrs and second session lasted 2.5hrs).	Community	Bilingual Nurse Practitioner (NP).	American Diabetes Association (ADA) and National Diabetes Education Program.	Pathophysiology of diabetes, complications, treatment modalities, medication, diet, exercise, self-management, self-monitoring of blood glucose and how to interpret results.	3 months
Fritschi et al. [18]	Technology-driven education. 2 in-person group sessions.	Clinic	DE, exercise physiologist.	Not reported.	Healthy eating and exercise.	6 weeks.
Hunt et al. [33]	Technology-driven education.	Community	Self-directed.	American Association of Diabetes Educators (AADE).	Blood glucose monitoring, eating healthy, taking medications, monitoring complications, and exercising.	Not reported.
Kellow et al. [34]	Culturally tailored education. 5 sessions 2hrs per session.	Primary care	Bilingual facilitator and multidisciplinary clinicians.	American Association of Diabetes Educators (AADE).	The American Association of Diabetes Education AADE7 Self-Care Behaviours.	6 months.
Kjellsdotter et al. [61]	Group-based education. 5 group sessions, 2hrs per session.	Primary care	Diabetes nurse.	Taking charge of one’s life with T2D model.	My idea of the illness T2D, Who am I-Who am I with T2D? Past–Present–Future, Obstacles, Opportunities, Strengths, Weaknesses, My goals, Challenges—my own learning process.	Not reported.
Knott et al. [32]	Structured patient education.	Transition from Hospital-based to Primary care	Nurse educator and dietitian.	Not reported.	A simple explanation of diabetes (symptoms), reassurance, self-monitoring, measurements, focus on food, and focus on feet.	Ongoing.
Knox et al. [35]	Peer mentoring program. 8-week series; large-group sessions and follow-up small group mentoring session.	Primary care	Peers.	Carpeta Roja Mentoring Program.	Not reported.	4 months to 2 years.
Liu et al. [37]	Structured patient education. 3 or 4 group sessions of 2.5hrs.	Primary care	Registered Nurses (RNs) and RDs and DE.	American Association of Diabetes Educators (AADE).	Healthy eating, exercise, acute and chronic complications, problem solving, goal setting, and ongoing support planning.	6 months.
Liu et al. [52]	Structured patient education—Individualized. One session.	Community	RNs.	Traditional principles of diabetes education.	Hypoglycemia education.	18 months.
Markle Reid et al [14]	Client-driven and community-based SM program. 3 in-home visits by RN, RD or both, six monthly group sessions.	Primary care	RNs, RD, program coordinator and peer volunteers.	Social Cognitive Theory. Empowerment.	Client-driven and flexible.	6 months.
Marsh et al. [36]	Individualized structured, with a focus on social determinants of health (SDH). 1 hr in home visits by CHWs and telehealth consultation with HCPs.	Primary care	Community health workers (CHW).	Translation framework & Iowa Model of Evidence-Based Practice.	The Association of Diabetes Care & Specialists (ADCES7) self-care behaviours curriculum, including healthy coping, monitoring, dietary habits, physical activity, taking medication, reducing risk and problem solving.	3 months.
Miklavcic et al. [13]	Client-driven and community-based SM program. 3 in-home visits by RN, RD or both; 6 monthly group sessions.	Primary care	RNs, RDs, program coordinator and peer volunteers.	Social Cognitive Theory. Empowerment.	Client-driven and flexible.	6 months.
Miller et al. [63]	Structured patient education. 10 weekly group sessions each session lasting 1.5- 2hrs.	Community	RD.	Theory of Meaningful Learning, the Information Processing Model, and Social Cognitive Theory.	Nutrition information (e.g., reading labels, tips for saving money).	10 weeks.
Miller et al. [31]	Structured patient education. 6-hr group education sessions.	Community	DESMOND’s trained and certified HCPs.	Diabetes Education and Self-Management for Ongoing and Newly Diagnosed (DESMOND) program.	DESMOND curriculum.	Not reported.
Narindrarangkura et al. [55]	Structured patient education (self-directed approach).	Clinic	No facilitator—self-directed.	SEE-Diabetes (Support-Engage-Empower-Diabetes).	Not reported.	Not reported.
Pauley et al. [25]	Coaching program led by paraprofessionals. Six 1-hr one-to-one in-home coaching sessions.	Community	PSWs.	Not reported.	Control group (CG): signs of hyper/hypoglycemia, skin care, insulin injection, and glycemia monitoring. Experimental group received CG education and priority goals-driven education.	6 weeks.
Redmond et al. [56]	Structured patient education. 8 sessions.	Community	Nutritionist.	The National Standards for Diabetes Self-Management Education.	Carbohydrate counting, portion control, meal spacing, physical activity, foot care, diabetes complication, and monitoring of blood glucose and AC1.	5 months.
Rygg et al [64]	Structured patient education. 15hrs over 3 group sessions.	Clinic	DE.	Not reported.	Lectures with introductory information and questions; interactive learning/skills training (physical activities, measuring blood glucose and problem-solving exercises; group discussion around the patient’s experience and questions arising in the group.	12 months.
Sanchez [26]	DSME support and goal-setting sessions.	Primary care	Physician and 2 NPs.	Plan-Do-Study-Act cycle, Chronic Care Model and principles of DSME.	Nutrition, signs and symptoms of acute and chronic complications, medications, and behavioural coping.	2 months.
Simmons et al. [27]	Peer support program. One session (3.5hrs) for group session, including peers and participants. Peer meetings = 1:1 meeting (1.5hrs).	Community	RD, nurse educators (training sessions to peers) and peers.	Not reported.	Identifying carbohydrates and understanding portions; truths and myths about diabetes; know your numbers and medications; and keeping active and looking after your feet.	2 months.
Smith et al. [54]	Group education sessions and nurse-client individual sessions. 3-hr introduction to T2DM, and refresher session.	Primary care	Nurses.	Not reported.	Client-centred and flexible.	Not reported.
Suhl & Bonsignore [22]	Individualized education.	Not reported	Not reported.	AADE & American Geriatric Society (AGS).	DE content was based on the AADE and the AGS standards of care.	Not reported.
Sullivan et al. [28]	Coaching program (self-directed approach) 4 follow-up telephone visits.	Clinic	Case manager.	Bandura’s Self-Efficacy Theory.	Self-management priority goals.	2 months.
Van Vugt et al. [30]	Structured patient education. 2 group meetings.	Primary care	DE.	The PRISMA (Dutch version of DESMOND) program.	T2DM, treatment, hyper-hypoglycemia, monitoring blood glucose, physical activity, complications and risks, medical outcomes measures, action planning and goal setting.	3 months.
Walter et al. [29]	Individual education. One 3-hr session.	Community	Pharmacist.	The Conversation Map program. Empowerment philosophy.	Topics discussed included the progressive nature of diabetes, diabetes ABCs (A1C, blood pressure, and cholesterol), short- and long-term complications of diabetes, lifestyle interventions (diet and physical activity), and medications used to manage diabetes.	Not reported.
Whitehouse et al. [57]	Structured patient education. Patient need determined the duration of each session.	Clinic	2APRN and DE.	American Association of Diabetes Educators (AADE).	DE content was based on the AADE and the ADA standards of care.	12 months.
Whitehouse et al. [12]	Technology-driven program. 4 week (30–60 minute weekly) individual synchronous education session.	Community	APRN and DE.	American Association of Diabetes Educators (AADE).	DE content was based on the AADE and the ADA standards of care.	3 months.

**Table 3:** Outcomes of DSME programs/interventions.

Author, Year	DSME Outcome Measures				Results of Outcomes
	Learning Outcomes	Behavioural Outcomes	Clinical Outcomes	Other Outcomes	
Akhter et al. [9]	Diabetes knowledge			Satisfaction	60% of participants increased their correct answers by at least one question. Those taking insulin increased the proportion of correct knowledge by five (95% confidence interval: 3–8), while others increased theirs by 10 (95% confidence interval: 9–12). In logistic regression, those increasing their correct answer by ≥10% had a lower proportion of correct answers at baseline, but no other variables showed significant associations. A total of 93.5% said the session met their expectations.
Andrich et al. [10]		Health care use, Quality of Life (QoL)	FBG		Use of Diabetes Self-Management Education and Support (DSME/S) was 20% prior to the intervention. Compliance increased to 35% after the DSME/S practice change initiative. This increase did not meet the objective of increasing utilization to 50%; however, the increase was statistically significant (*p* < 0.05). The decrease in mean FBG levels pre- and postintervention was statistically significant (*p* < 0.05). QoL improved following DSME/S. Reinforce DSME after diagnosis and follow-up.
Babalola et al. [15]	Diabetes knowledge	Dietary habits, physical activity, health care use and blood glucose management (BGM)	HbA1C		There was a significant improvement in pre-education and post-education outcomes: a) knowledge, t(11) = −7.969, p = 0.000; d = 2.32, b) self-management, t(11) = −7.930, p = 0.000; d = 2.43, and c) HbA1C levels, t(11) = 6.434, p = 0.000; d = 0.78.
Bastiaens et al. [60]		Dietary habits, physical activity and emotional distress.	HbA1C, BMI		BMI decreased modestly by 0.45 kg/m2 (95%CI 0.01–0.89) (*N* = 35) at 12-month and by 0.53 kg/m2 (95%CI 0.02–1.04) (*N* = 32) at 18-month follow-up. Mean HbA1C declined from 7.4% (±1.3) to 6.8% (±0.8) (p = 0.040). The Problem Areas in Diabetes (PAID)-score diminished from 28 (±20) to 18 (±13) (p = 0.006) at 12-month follow-up. At 18 months post-intervention no significant difference was seen. Emotional distress improved significantly after 12 months only (10 points). Effects on actual behaviour were based on observations but could not be confirmed based on the food frequency and IPAQ questionnaires.
Bowman & Epp [65]	Diabetes knowledge & self-efficacy	QoL and health care utilization			Mean scores were higher for Group A (attendees) than for Group NA (non-attendees) (53.8, *SD* = 27.3; 39.6, *SD* = 26.1, respectively), approaching statistical significance (Mann-Whitney U = -1.904, *P* = .057). Mean scores at Site 1 (52.2; *SD* = 28.5) differed from those at Site 2 (57.7; *SD* = 24.9). Diabetes Care Profile (DCP) education, understanding, and PAID scores did not differ by group status, although PAID scores were somewhat higher for Group A than for Group NA. PAID scores differed by age level (Kruskal-Wallis *c*^2^ = 19.160, *df* = 3, *P* < 0.001), 43 to 59 years (26.0; *SD* = 26.3) compared to those aged 60 to 69 (12.5; *SD* = 20.5), 70 to 79 (10.8; *SD* = 9.0), or 80 or older (7.5; *SD* = 10.3). Mean PAID scores were higher for women than for men (Mann-Whitney U = -2.603, *P* = 0.009). Satisfaction with care was significantly higher in Group A than Group NA (Mann-Whitney U = -3.646, *P* < 0.001). QoL scores were higher among attendees. Group NA tended to use the ER (for all reasons) more than Group A (approximately 2:1) and had increased short-stay hospitalization utilization (for all conditions).
Braun et al. [16]	Diabetes knowledge	QoL, Glucose monitoring, insulin injection	Cognitive function, HbA1C	Satisfaction	The mean cognitive function of all 102 patients was 87.7 ± 12.3 IQ points at enrolment and decreased in comparison with the premorbid cognitive ability 96.3 ± 9.2 IQ points (P <0.001), reflecting the decrease of intelligence. After TTP there were no differences in knowledge and ability for DSM (standard/DICOF: knowledge 11.0±2.6 vs. 12.2± 2.7 points, P = 0.11; handling 14.9± 3.3 vs. 15.9±2.5 points, P = 0.18). However, patients who took part in the DICOF programme showed better scores in satisfaction with the education programme [standard/DICOF 44.7 (31–57) vs. 52.5 (45–59) points, P < 0.001]. Six months later, the DICOF participants showed better results regarding DSM (standard/DICOF: handling 12.5± 4.1 vs. 15.9± 3.1 points, P = 0.001). Both groups showed HbA1c decrease (8.3± 1.4 vs. 8.5±1.3%, P = 0.62) and similar incidence of acute complications.
Braun et al. [62]		QoL			Only patients switched on insulin therapy showed significant improvement in diabetes-related QoL 6 months after participation in the DTTP (p = 0.03), fewer physical complaints (p = 0.03), fewer worries about the future (p = 0.02), fewer daily struggles (p = 0.01) and less fear of hypoglycemia (p < 0.001), while patients who were already on insulin therapy showed no improvements in diabetes-related QoL.
Braun et al. [38]	Diabetes knowledge	BGM, insulin injection	HbA1C		SGS participants showed improved levels of HbA1C 6 months after the DTTP, and less acute complications than the standard group (P<0.009). Both groups demonstrated a good capacity for DSM and improvement in diabetes knowledge after the DTTP (P<0.01).
Choi et al. [59]		Dietary habits, physical activity, BGM food care and medication, and depression.	HbA1C, Waist circumference (WC), lipid panel		From baseline to 3-month follow-up assessment, participants exhibited significant improvement on several physiological and behavioural measures. Clinical measures such as HbA1C decreased from baseline to 3-month follow-up: 7.3 to 6.8% [*t*(39) = 5.13, p < .001] and WC also decreased: 38.5 to 37.3 inches [*t*(40) = 4.89, *p <* .001]; while HDL levels increased 44.1 to 47.8mg/dL [*t*(36) = −3.52, p < .01]. Diabetes management behaviours, number of reported feet checks per week increased across the three assessments 1.7 to 2.8 to 3.1 times [*F* (2,80) = 12.70, p < 0.001], and there was a trend increase in participants’ reported frequency of exercise activities [*F*(1.71, 68.37) = 2.88, *p<*. 10]. The health and well-being variables showed no significant change across the three assessments.
Gorter et al. [24]				Preference	Eighty per cent of respondents preferred diabetes education during regular diabetes check-ups. Patients taking insulin preferred education to be given by nurses [odds ratio (OR) 2.45; 95% confidence interval (CI) 1.21–4.96]. Individuals who thought their health to be poor ⁄ average preferred education to be given by doctors (OR 1.65; 95% CI 1.08–2.53). Physical exercise was the preferred self-care activity of those who thought they took too little exercise (OR 1.97;95%CI 1.32–2.93) but was preferred less by patients with mobility problems (OR0.65;95%CI 0.43–0.97). Patients with eating disinhibition reported keeping to a healthy diet (OR4.63; 3.00–7.16) and taking medication (OR1.66;95% CI 1.09–2.52) as the most burdensome self-care activities. Age was not an independent determinant of any preference.
Hunt et al. [33]	Diabetes knowledge				There was a statistically significant difference in pre-intervention knowledge and post-intervention knowledge scores (t = 10.94, p = <0.0001).
Kellow et al. [34]		Dietary habits, physical activity, BGM, medication adherence, problem solving, healthy coping and reducing risks, mental distress	HbA1C, lipid profile, WC, waist-to-hip ratio (WHR)		At program completion, mean participant WC (90.5 versus 89.2 cm, p < 0.001) and WHR (0.574 vs. 0.566, p < 0.001) was significantly reduced and both were further reduced at 6-month follow-up (p < .05). There were no significant changes at baseline and at 6-month follow-up in mean HbA1C (*p* = 0.32), total cholesterol (*p* = .78), HDL (*p* = 0.37) and TG (*p* = 0.84). There was a significant increase in the median frequency of diabetes self-care behaviours undertaken, with American Association of Diabetes Educators Questionnaire Score: 30 (22–32.3) versus 33 (29.8–35.0), p < 0.001 at 6-month follow-up. Diabetes-related distress assessed by PAID-C was also significantly reduced at 6-month follow-up (p < 0.05). Mean HbA1C was unchanged after 6 months; 51 (7.9) versus 50 (7.8) mmol/mol, p = 0.316. DSME interventions/programs must consider culture, preference for information, health literacy, and disabilities.
Kemper et al. [19]	Diabetes knowledge	Health care use, BGM			The group with high school diplomas or GEDs reported more diabetes knowledge about the cause of low blood sugar (*M* = 3.55, *SD* = 1.21) than the group who did not finish high school (*M* = 2.29, *SD* = 1.45). This difference was significant, *t*(26) = –2.374, *p* < .05 (two-tailed). The high school graduates also reported more diabetes education about the treatment of low blood sugar (*M* = 4.00, *SD* = .89) than the group who did not complete high school (*M* = 2.71, *SD* = 1.57). This difference was significant, *t*(26) = –2.474, *p* < .05 (two-tailed). 26 participants owned a glucometer. 17 (60.7%) participants checked their blood sugar at least once a day; 8 participants (28.5%) used the glucometer 1 to 3 days per week. 60% documented their blood sugar results in a log book. Only 1 person did not use a glucometer at all. 13 persons (46.4%) had seen their primary care physician for their diabetes once in the past 3 months, 4 (14.3%) twice, 1 (3.6%) three times, and 1 seven times. Only 1 participant had been hospitalized for diabetes in the past 3months.
Knott et al. [32]			HbA1C, BMI, Lipid and renal profiles, BP		There were no significant differences between locations in the reduction in HbA1C. The changes in body mass index (BMI) observed between diagnosis and 3 months in all three settings were also similar. Similar changes in lipid profile, renal function and blood pressure were also observed over the same period (2003–2004). A well-established education program has successfully transferred to the community without affecting clinical outcomes.
Knox et al. [35]	Diabetes knowledge	Dietary habits, BGM, medication adherence	HbA1C, LDL, BP, BMI		Both intervention and control groups showed significant improvement on all health indicators from baseline to 6-month follow-up (*P* < .001). HbA1C decreased slightly faster for patients in the intervention group (*P* = .04). SMB improved significantly from baseline to 6-month follow-up for the intervention group. Interviewed participants also reported reductions in social isolation and extension of impact of health behavior changes to multiple generations of family members.
Liu et al. [37]		Health care use	HbA1C, Lipid profile, Creatinine, eGFR, Blood pressure (BP), BMI		HbA1C decreased by 1.2 percentage points (95% CI 0.9–1.6, *P* < 0.001). BMI decreased by 0.7 kg/m2 (95% CI 0.4–1.0, *P* < 0.001). After the intervention, participants had an average decrease in systolic blood pressure of 2.7 mmHg (95% CI 0.3–5.1, *P* = 0.03) and improvements in their lipid profile, with an average decrease in total cholesterol of 6.9 mg/dl (95% CI 1.5–12.4, *P* = 0.01). Changes in creatinine and estimated eGFR were not statistically significant. An additional 15.9% of participants received eye exams, 22.7% received foot exams, and 41.4% were evaluated for microalbuminuria. All these changes were significant at *P* < 0.001. At baseline, 82.3% of patient had ophthalmological examination, 47.7% food examination and urine checked for microalbuminuria. After the intervention, and additional 15.9% of participants received foot exams, and 41.4% were evaluated for microalbuminuria. All these changes were significant at *P* < 0.001.
Liu et al. [52]		Health care use			Overall, the participation rate of diabetes self-management education was 46.8% (95% CI: 44.4%-49.2%). The rate was 40.3% for beneficiaries in rural areas, 48.0% for suburban areas, and 47.3% for urban areas. About 31% of beneficiaries newly diagnosed with diabetes did not participate within the past year. Those who were older, with lower education, and lower income levels were less likely to have participated (*P* < .05).
Markle Reid et al. [14]	Self-efficacy	QoL, mental functioning, dietary habits, physical activity, foot care		Cost	Morbidity burden was high (average of eight comorbidities). Intention-to-treat analyses using analysis of covariance showed a group difference favoring the intervention for the MCS (mean difference = 2.68, 95% confidence interval (CI) = 0.28–5.09, P = .03), SDSCA (mean difference = 3.79, 95% CI = 1.02–6.56, P = .01), and CES-D-10 (mean difference = 1.45, 95% CI = 0.13 to 2.76, P = .03). No group differences were seen in PCS score, anxiety, self-efficacy, or total health care costs. The 6-month program reduced symptoms of depression in the intervention group.
Marsh et al. [36]	Diabetes knowledge	Dietary habits, physical activity, BGM, foot care, smoking)	HbA1C	Satisfaction	The median hemoglobin HbA1C level of the patients at baseline was 9.2 (interquartile range [IQR] 2.8), and the median A1C level at follow-up was 7.2 (IQR 2.4), suggesting a statistically significant A1C reduction (Z = 22.31, *p* = .021). The Cohen *d* was 0.90, indicating a large effect size. The Wilcoxon signed-rank test revealed a statistically insignificant change in the SDSCA median sub-scores before and after project completion (*p* > 0.05). There was a statistically significant increase in diabetes knowledge among older adults (*Z* = 2.94, *p* = 0.003). The Cohen *d* was 2.28, indicating a large effect size.
Miklavcic et al. [13]	Self-efficacy	QoL, mental functioning, dietary habits, physical activity, foot care		Cost	No significant group differences were seen for the baseline to six-month change in physical functioning (mean difference: -0.74; 95% CI: − 3.22, 1.74; p-value: 0.56), mental functioning (mean difference: 1.24; 95% CI: − 1.12, 3.60; p-value: 0.30), or other secondary outcomes. Results were inconclusive.
Millar et al. [66]		BGM, medication adherence, health care use			Over 13% of the population with diagnosed diabetes attended a DEC in 1 year. People with recently diagnosed diabetes were more likely to attend. There was a strong association between attending a DEC and blood glucose self-monitoring, suggesting that diabetes education is effective to increase self-care behaviour. The secondary outcomes were also associated with diabetes education, suggesting patients receiving diabetes education may become empowered to more effectively advocate for appropriate processes of care from their treating doctors.
Miller et al. [62]			FPG, HbA1C, Lipid profile		Participants exceeded the guidelines for optimal glycemic control at pretest. The experimental group had greater improvements in fasting plasma glucose (P = 0.05) and glycated hemoglobin (P < 0.01) than the control group. Significantly more participants in the experimental group than control group met the treatment goals for total cholesterol at posttest (P < 0.05).
Miller et al. [31]	Diabetes knowledge				Patient activation significantly increased by 9.7 points from pre to post DESMOND intervention (z = –7 .94, p < 0.001). Of all participants who exhibited an increase in patient activation, 87% (n = 142) experienced a clinically significant (>5 point) increase. Post-DESMOND participation, an 86% reduction (from 6% to 0.9%) in the proportion of participants scoring in the lowest PAM level (Level 1) was observed (p < 0.01).
Murray et al. [53]		Medication adherence, health care use.			Patients attending diabetes education programs had greater utilization of statins (70.6%) than non-attendees (69.4%, p < 0.0001). Diabetes education program attendance was also associated with greater utilization of glucose lowering medications (83.7%vs.82.0%, p < 0.0001), antihypertensive medications(90.2%vs.89.7%, p < 0.0001),angiotensin converting enzyme inhibitors/angiotensin receptor blockers(79.8%vs.78.9% p < 0.0001),and glucose monitoring strips (82.2%vs.65.6%, p < 0.0001);and visits to ophthalmology/optometry (78.7% vs. 72.7%, p < 0.0001).
Pauley et al. [25]	Self-efficacy	Anxiety and depression			Both groups showed improvement in Diabetes Self-Efficacy Scale (DSES) (6.6 + 1.5 vs. 7.2 + 1.5, p < 0.001) and Insulin Management Diabetes Self-Efficacy Scale (IMDSES) (113.5 + 20.6 vs. 125.7 + 22.3, p < 0.001); there were no between-groups differences. There were no between-groups differences in anxiety (p > 0.05 for all) or depression scores (p > 0.05 for all), or anxiety (p > 0.05 for all) or depression (p > 0.05 for all) categories at baseline, postintervention, or follow-up.
Redmond et al. [56]	Diabetes knowledge	Dietary practices, physical activity, BGM, foot care	HbA1C		HbA1C levels significantly decreased by 0.66 and 1.46% among those with pretest HgA1C of > 6.5% and > 8%, respectively (P ≤ 0.01); compliance significantly increased following a healthful diet, following an eating plan, avoiding high fat foods, spacing carbohydrates, testing blood sugar as recommended by health care provider and inspecting shoes (P ≤ 0.05); the number of HbA1C knowledge questions answered correctly increased from 42% to 65% (P < 0.0001); decreases in HbA1C among those with an initial HbA1C > 6.5% were correlated with increases in physical activity (P ≤ 0.05).
Rygg et al. [64]	Diabetes knowledge	QoL	HbA1C, BMI, Lipid profile, Creatinine		There were no differences in the primary outcomes between the groups at 12 months, but the control group had an increase in HbA1C of 0.3% points during follow-up. Diabetes knowledge and some self-management skills improved significantly in the intervention group compared to the control group. A subgroup analysis was conducted for the quartile with the highest HbA1C at baseline (>7.7, n = 18 in both groups). There were significant improvements within the intervention group at 12-month follow-up for both HbA1C and PAM and a trend for better outcome in the intervention group compared to the control.
Sanchez [26]			HbA1C, BP, Lipid profile	Cost	Shared medical appointments (SMA) improved HbA1C, self-management skills, and satisfaction. A case for financial viability of the continuous quality improvement (CQI) project was evident in the billing and reimbursement process.
Shah et al. [11]		Health care use			Self-management programme attendees were more likely than non-attendees to achieve process measures of quality of care such as retinal screening examinations (75.3% versus 70.3%, adjusted relative risk 1.05, 99% confidence interval 1.03–1.08), and ≥2 glycated haemoglobin tests (57.5% versus 53.3%, adjusted relative risk 1.08, 99% confidence interval 1.05–1.11). However, with a median follow-up of 5.3 years, diabetes complications and mortality were not different between arms.
Sullivan et al. [28]		Adherence to medication, physical activity, BGM, health care use			Twenty-eight days after discharge, 6 participants verbalized increased in physical activity, five reported an improvement adjustment in eating habits and medication adherence, 4 reported improved BGM, although only 58% recorded daily readings. Ninety-five percent reported scheduling a primary care provider appointment; 68% made the visit. Four of 20 patients were readmitted to the acute care setting ranging with an average of 18 days of inpatient stay.
Van Vugt et al. [30]		Illness perception, foot care, dietary habits			Improvements were found in illness perception (b = 1.586, p < 0.001),general diet (b = 1.508, p = 0.001),footcare(b = 0.678, p = 0.037),weekly average diet (b = 1.140, p = 0.001),creating action plan (b = 0.405, p = 0.007).Well-being interaction effects were found for general diet (p = 0.009),weekly average diet (p = 0.022), and creating an action plan (p = 0.002).
Walter et al. [29]	Diabetes knowledge	Attitude			A statistically significant improvement in attitude (P <0.05) was observed for most areas addressed by the pre-test/post-test. For most areas, the improvement was small. However, the greatest areas of improvement were seen in understanding non-drug actions for managing diabetes, understanding the purpose of medications, and believing that changes in daily life would improve overall health. There was not a statistically significant improvement (P = 0.13) in participants’ understanding of how to take medications correctly.
Whitehouse et al. [57]		Health care use	HbA1C		Rates of rehospitalization and HbA1C improved for older adults who received nurse-led inpatient DSME and homecare during transitions of care from hospital to home. Rehospitalization rates up to 90 days were decreased for the DSME plus homecare group (10%) compared to DSME only (20%) and usual care groups (26.7%) (p < 0.05). A decrease of −0.4 and −2.3 HbA1C units was observed for the DSME group and DSME plus homecare group, respectively, at 90 days.
Whitehouse et al. [12]	Diabetes knowledge	Health care use	HbA1C		Participants who completed the intervention general diabetes knowledge significantly improved from 63 (SD = 20) to 78 (SD = 14) (p = 0.02), as did knowledge of insulin, which improved from 61 (SD = 28) to 81 (SD = 17) (p = 0.02). HbA1C level improved from 9.5% (SD = 2.3) to 8.5% (SD = 1.7%) (p = 0.22), and there were no hospital readmissions for any patient who completed the program. Participants described the program as useful and were satisfied with the program.

### Review findings

#### Research sub-question one

How is DSME for older persons defined in the literature by study authors?

#### Definition of DSME in the literature by study authors

DSME was defined by researchers as an essential component of patient-centred care [9, 10, 20, 24, 36, 54] and a collaborative process used to facilitate the required knowledge, self-care practices, coping skills, and attitudes to modify behaviour and successfully prevent or delay complications [9, 12, 17, 23, 26, 30, 37, 53, 55–57]. DSME supports people with diabetes and their families [55], and it has been successful in reducing HbA1C levels [56] and improving behavioural and clinical outcomes in older populations [12, 22, 26, 30, 37, 53–57]. The goal of DSME is to support informed decision-making, self-care behaviours, problem-solving, and active collaboration with the health care team to achieve optimal health status and a better quality of life (QoL) [9, 10]. To be effective, the development of DSME must take peoples’ life experiences into account, including the social determinants of health (SDH) and evidence-based standards of care [17, 20, 23, 24, 29, 36, 58].

#### Research sub-question two

How is DSME defined by older persons?

#### Definition of DSME by older persons

Of all 44 papers, one had a definition of DSME by older persons with diabetes. In the study by Robertson [20], participants’ understanding of diabetes education was shaped by their first engagement and interaction with other older persons with diabetes during their first sessions after diagnosis.

#### Review sub-question three

What types of DSME programs have been reported in the literature for older persons with diabetes?

#### Types of DSME programs

The education programs differed in their design, including strategies used, delivery mode, theoretical underpinnings, and duration. Tables 2, 3 are the detailed characteristics of all DSME programs and related study components. Thirty-two (76%) studies had the type of education, including empowerment models [9, 17], support and goal-setting programs [10, 26], culturally-tailored education [15, 34, 59], group-based programs [54, 60, 61], structured programs [16, 30–32, 37, 38, 52, 56, 57, 62–64], technology-driven education [12, 18, 33, 55], peer mentoring programs [27, 35], client-driven and community-based self-management (SM) programs [13, 14], coaching programs [25, 28], and individualized education [22, 29, 36].

A diverse group of theories and frameworks guided the development of the DSME interventions/programs: the health promotion model [15]; the Plan-Do-Study-Act cycle [10, 26], which includes a Plan phase wherein the objective for implementation is decided, a Do phase for identification and scheduling of patients, a Check [Study] phase wherein charts are reviewed, and workflow and no-show rates are assessed, and Act phase to start the planning cycle again based on identified barriers and interventions [10, 26]; empowerment philosophy [9, 17, 29–31, 55, 60]; trans-theoretical model [60]; social cognitive theory, which recognizes the central role of self-efficacy in achieving self-management behaviour (SMB) change [13, 14, 30, 31, 60]; behavioural change and motivational technique [60]; Bandura’s self-efficacy theory [28]; theory of meaningful learning [63]; the information processing model, which outlines the steps used to acquire and process information leading to a decision [63]; Leventhal’s common sense model of illness, which focuses on an individual’s illness representation or personal model of diabetes as a key determinant of an individual’s behavioural and emotional response to illness [31]; chronic care model, which creates practical, supportive, evidence-based interactions, shared medical appointments, and principles of DSME [26]; taking charge of one’s life with T2D model [61]; Carpeta Roja, developed by Latino Health Access in Santa Ana, California [35]; traditional principles of diabetes education [27, 52]; programs based on the American Diabetes Association (ADA) and National Diabetes Education Program [59]; translation framework and Iowa Model of Evidence-Based Practice [36]; and the American Association of Diabetes Educators (AADE) [12, 33, 34, 37, 55, 57].

In 34 studies, the topics of education were reported, including the basics of diabetes mellitus [29, 30, 31, 35, 59, 61, 62], dietary habits [9, 10, 12, 15, 16, 18, 22, 26, 27, 30–34, 36, 37, 56, 57, 60, 63], blood glucose monitoring [9, 10, 16, 25, 27, 29–33, 36, 38, 52, 56, 59, 62, 64], available treatments [9, 16, 25–27, 29–31, 33, 38, 59], physical activity [9, 12, 15, 18, 22, 27, 30, 31, 33, 34, 36, 37, 56, 57, 59, 60, 64], diabetes-associated complications [12, 15, 16, 22, 25, 26, 29–31, 33, 34, 36–38, 52, 56, 57, 59, 62], foot care [9, 27, 32, 56], health care use [10, 12], goal-setting behaviours [12, 15, 22, 25, 28, 30, 31, 34, 37, 57, 61], behaviour and healthy coping [26, 36], psychosocial support [10, 12, 15, 22, 34, 37, 57], identification of barriers to diabetes self-care behaviour [12, 15, 22, 57, 61], lifestyle modification [10, 29], problem-solving [12, 15, 22, 34, 36, 37, 57, 64], and client-driven and flexible education [13, 14, 17, 54, 64].

Researchers described various methods of engagement in DSME for older persons, specifically: culture circles, group discussion, quizzes, and experiential learning [9, 13, 14, 17, 32, 64]; in-person or virtual peer-to-peer mentoring sessions [29, 35]; exercise sessions and a light meal [13, 14]; a supermarket session (as part of several sessions) to facilitate the application of principles learned during the intervention [63]; developing a lesson plan and handouts, including tips on how to manage diabetes [56]; biweekly community health worker (CHW) visits to evaluate SDH and telemedicine to reinforce diabetes self-care activities for older persons living on a low income [36]; measuring blood glucose during the session [64]; video, handouts, oral presentations, and bilingual education [26, 34, 59]; and individual synchronous conversations [12].

#### Review sub-question four

What types of research designs have been used to understand the development, implementation, and evaluation of DSME programs for older persons?

#### Research designs

Of all 44 papers, 32 (70%) had a quantitative design, the majority of which were one-group pre-test and post-test studies (n = 13; 30%), followed by RCTs (n = 7; 16%) and static group comparisons (n = 4; 9%), while only three were qualitative (7%), two were quality improvement projects (5%), and three followed a mixed-methods design (7%). In four papers the research design was not reported or not applicable [17, 22, 23, 32].

#### Review sub-question five

In what clinical (e.g., primary care, acute care) contexts have DSME programs been developed?

#### The context of DSME programs

Most of the studies were developed in community settings (n = 12), followed by programs in primary care (n = 11) and hospitals (n = 10). One program was described as a successful transition from hospital-based to primary care [32]. Only four studies made education available to providers, including in-service education on DSME and the ADA Standards of Medical Care in Diabetes algorithm [10] and formal training for professionals [34] and paraprofessionals [25, 36]. In most studies, nurses (e.g., registered nurses [RN], nurse practitioners [NPs], and nurse educators) (43%) were facilitators of the programs, followed by diabetes educators (16%) and registered dietitians (16%). In four studies (9%) peers were involved in the programs/interventions [13, 14, 27, 35], and in two studies (5%) there was a self-directed approach to education [28, 55]. The lengths of the programs varied; however, most programs lasted 6 months (20%), 3 months (20%), or 2 months (7%). There was diversity in the number of sessions in the programs or interventions; most education occurred in one session (14%) or five sessions (14%).

#### Review sub-question six

What outcome measures have been used to determine the effectiveness of DSME?

#### Outcomes of the DSME programs

Study outcomes are summarized in Table 3. Of 44 articles, 34 included learning, behavioural, clinical, and other outcomes.

*Learning outcomes*. Two indicators of learning outcomes were assessed in 18 studies: diabetes knowledge and self-efficacy (i.e., belief of confidence in one’s self-management). In 13 studies, researchers measured the effect of DSME programs/interventions on diabetes knowledge. Of those studies, 11 had improvements in diabetes knowledge [9, 12, 15, 29, 31, 33, 35, 36, 38, 56, 64], one had no changes in knowledge [16], and in one it was concluded more education did not translate to greater diabetes knowledge [65]. Self-efficacy was measured in four studies: in two studies there were improvements in self-efficacy scores [25, 65]; in one study there were no reported differences in self-efficacy after the intervention [14]; and results were inconclusive in another study [13].

*Behavioural outcomes*. Behavioural outcomes were presented in 26 studies. These behavioural outcomes included self-management behaviour (SMB), mental health, and quality of life (QoL) indicators. In 21 studies, the SMB indicators included dietary habits, physical activity, health care use, blood glucose monitoring, medication adherence, skills in the administration of insulin, foot care, problem-solving, healthy coping, and reducing risks. Of these studies, 19 had improvements in SMBs [11, 12, 14–16, 29, 30, 34–38, 53, 56–59, 65, 66]. In one study, conclusions about the effects on SMB were based on observations and could not be confirmed based on assessment tools [60]. In one study, the results for behavioural outcomes were inconclusive [13].

Six studies had mental health indicators, including the assessment of mental distress, anxiety, and depression [13, 14, 25, 34, 59, 60]. In two studies the authors reported no difference in anxiety and depression scores for participants following the intervention [25, 59]. In three studies, the researchers described how the education program reduced symptoms of depression in the intervention group [14, 34, 60], and in one study, the results for mental health outcomes were inconclusive [13].

QoL was measured in seven studies [10, 13, 14, 16, 62, 64, 65]. Of these studies, five had a statistically significant increase in QoL scores [10, 14, 16, 62, 65], one had a trend of worsening mental health QoL [64], and in another, the QoL results were inconclusive [13].

*Clinical outcomes*. Clinical outcomes included fasting blood glucose (FBG), HbA1C, body mass index (BMI), cognitive function, blood pressure (BP), waist circumference (WC), waist-to-hip ratio (WHR), lipid profile, and renal profile.

HbA1C, the most common outcome, was measured in 16 studies, of which 11 reported a significant improvement following the intervention [12, 15, 26, 35–38, 56, 57, 59, 60, 63, 64]. For example, Choi and Rush [59] reported a decrease in HbA1C levels from baseline to the 3-month follow-up: 7.3 to 6.8% (t[39] = 5.13, p < 0.001). In one study HbA1C decreased from 7.4% (±1.3) at the 12-month follow-up to 6.8% (±0.8) (p = 0.040) at the 18-month follow-up [60]. In another study it was determined, to improve learning outcomes, HbA1C should be controlled in older persons with diabetes and impaired cognitive function before participation in DSME [16]. In two studies, there was no significant reduction in HbA1C following the intervention. Kellow et al. [34] found no significant changes in mean HbA1C between baseline and the 6-month follow-up (p = 0.32). Similarly, Knott and colleagues [32] determined transferring a DSME program from a hospital-based setting to three community settings did not affect HbA1C outcomes.

The other clinical outcomes included the following. Positive changes in FBG were described in two studies [10, 31]. Changes in weight or BMI were measured in four studies [32, 35, 60, 64]; in two of those studies, the authors reported statistically significant positive changes [35, 60], and in the other two studies researchers reported no differences in BMI [32, 64]. Cognitive function was measured in one study, wherein they demonstrated the effectiveness of DSME in older persons with diabetes and impaired cognitive function [16]. BP was measured in three studies [26, 35, 37]; in two of the studies they reported a significant decrease in BP [35, 37], and in the third they reported BP did not change during the intervention [26]. Lipid profiles were measured in eight studies [26, 32, 34, 35, 37, 59, 63, 64]; in three studies they reported significant improvements in lipid profiles [35, 37, 63], and in five they reported no statistically significant changes [26, 32, 34, 59, 64]. WC was measured in two studies and in both it decreased following the intervention [34, 59]. In one study they reported a significant reduction in waist-to-hip ratio (WHR) at program completion [34]. Renal profile was evaluated in three studies, wherein there were no significant changes following the intervention [32, 37, 64].

*Other outcomes*. Health care cost was mentioned as an outcome in three studies [13, 14, 26]. In one study the authors detailed the financial viability of the project in the billing and reimbursement process [26] and in another study they noted no changes in total health care costs during the intervention [14]; in the third study health care costs were inconclusive [13]. In three studies satisfaction was an outcome, and in all three the scores were high [9, 16, 36]. In Gorter et al.’s [24] study, education preference was assessed as an outcome; 80% of participants preferred diabetes education during regular check-ups, and participants taking insulin preferred receiving education from nurses (odds ratio [OR] 2.45; 95% confidence interval [CI] (1.21–4.96). Individuals who considered their health to be poor/average preferred to receive education from doctors (OR 1.65; 95% CI 1.08–2.53).

#### Review sub-question seven

What are the gaps in the literature, including those identified by researchers, related to the development, implementation, and evaluation of diabetes self-management education for older persons living with diabetes?

We identified five gaps in the literature related to DSME for older persons in Western countries: the dearth of participation of older persons in DSME; limited or unclear minority representation; lack of qualitative research approaches; limited description of the sustainability of programs; and scarce description of available education for providers.

#### Gap 1. The dearth of participation of older persons in DSME

How DSME is defined and implemented is critical to improving diabetes prevention and management, and this should be guided by the ultimate goal of diabetes education—to control one’s diabetes through the development of their skills and knowledge to facilitate daily living, improve clinical and behavioural outcomes, and prevent complications [9, 10, 20]. DSME definitions and implementation are mediated through disciplinary and theoretical lenses to assist practitioners in the design, development, implementation, and evaluation of DSME programs.

There were older persons’ definitions of diabetes education in one qualitative study [20]. To implement DSME, most studies were developed by multidisciplinary teams, including endocrinologists, NPs, RNs, CHWs, general practitioners, psychologists, physical activity counsellors, dietitians, psychiatrists, diabetes educators, physiotherapists, podiatrists, and nutritionists [15, 25, 34, 36, 52, 56, 60]. In one study, the modules for the intervention were developed by nursing faculty and students with support from a computer science and software engineering department [33]. For culturally diverse groups, bilingual professionals were included in the team [15, 34, 59], and in one study, the team included paramedics and emergency staff [52].

The inclusion of older persons, family caregivers, and community service providers in the development of the DSME program was limited [13, 14]; in two programs, peer mentoring was at the centre of the process of education [27, 35]. This work has been important, useful, and necessary; however, what remains strikingly lacking is understanding the insights of and developing DSME programs with the individuals most impacted by the phenomenon—older persons living with diabetes. Such experiential knowledge is vital in the development of educational programs to ensure alignment with the preferred learning styles, literacy levels, culture, and needs of this population—an approach that could manifest more substantive, sustained results.

#### Gap 2. Limited or unclear minority representation

Race and ethnicity data were poorly described or lacking in most studies [10, 11, 13, 14, 16, 19, 24, 25, 27–29, 31–33, 38, 52, 53, 58, 60–62, 64, 66]. In seven studies, the population was described primarily as “white” [22, 35, 36, 54, 55, 58]; other terms used by researchers were “caucasian” [30, 56, 63, 65], “English ethnicity” [20], “Western ethnicity” [24], and “British” [9]. In the remaining five studies, the population was primarily described as “Black” [12, 37, 57] and “Mexican American” [15, 26]. There are well-defined inequities in which racialized and ethnic groups have reduced access to DSME [15, 19, 37].

Additionally, there is a need to include rural communities in the development of DSME programs. In this review, 10 studies included rural communities [9, 11, 13, 20, 33, 35, 36, 58, 65, 66]. Luo et al. [58] described how participation in diabetes education in rural settings was 7% lower than in urban areas. Robertson et al. [20] found rural living was described by participants as a barrier to successful diabetes management and education; the distance to access physical activity programs, groceries, diabetes education, and medical care was noted as a major concern. Furthermore, participants talked about fewer opportunities for DSME programs [20]. Rural communities are characterized by lower rates of personal income, educational attainments, health care access, access to healthy and affordable food access, and more environmental barriers—these factors must be considered when developing diabetes education for older persons in these communities [20, 65]. The development of DSME programs must involve evidence-based practices and address the unique barriers resulting from living in rural communities [20, 36]. A strategy to improve access to DSME, as suggested by Marsh et al. [36], includes the use of CHWs enrolled as peer coaches for patients with diabetes in rural settings. In their study, during the home visit, the CHWs performed routine SDH assessments and provided basic diabetes education. In addition, they facilitated telehealth consultations for patients with health care providers (HCPs). This intervention successfully bridged the effects of limitations in regard to the SDH for older persons with diabetes in rural settings, including the lack of internet, health literacy, and or reliable transportation that restricts their participation in DSME [36].

Socioeconomic status—a SDH—was reported in 13 studies [11, 13, 15, 18–20, 28, 35, 53, 54, 58, 59, 65]. Older persons with diabetes living on a low income are often underrepresented in the development of DSME strategies [18]. Kemper et al. [19] found that older persons without a high school diploma received less formal diabetes education than those with a high school diploma. To improve learning, clinical, and behavioural outcomes in diabetes, DSME programs must attend to the effects of the SDH on diabetes self-care practices for older persons [17]. Future work should clearly describe the race, ethnicity, income, type of diabetes and education level of research participants. In addition, efforts should be made to include other older persons with diabetes in circumstances of vulnerability, such as older persons with disabilities, older persons living on a low income, immigrants, refugees, and members of LGTBQ2S+ communities to participate in future DSME research and the design, development, implementation, and evaluation of programs.

#### Gap 3. Lack of qualitative research approaches

The use of qualitative approaches to study DSME for older persons is limited. Of the 44 studies, only six used qualitative research designs [20, 54, 61] or methods [35, 52, 55]. Qualitative research is essential for the development of well-rounded, meaningful, client-oriented DSME programs, as it enables a deeper understanding of experiences, phenomena, and context. Researchers have identified the high risk of complications in older persons, who often fail to obtain or retain self-management competencies [46]. For example, in the studies by Liu et al. [52] and Robertson et al. [20], older persons felt diabetes education was helpful during diagnosis but was less effective, highly controlling, and repetitive at later stages. In both studies, participants shared they did not need diabetes education [20, 52]. This is a finding that cannot be ignored and must be addressed through further investigation as to its meaning (such as whether there is full awareness of all the illness implications [regardless of lack/presence of symptoms], whether there is preference to seek information only when problems arise, and or whether content, structure of education or both have influence on this perspective), and to the development of strategies that guarantee continuous DSME for older persons to avoid complications. Through qualitative data, older persons can share their suggestions about strategies for organizing DSME programs in their communities. The value of group sessions was described in almost all studies [20, 35, 52, 54, 61], and the addition of peer mentoring programs as a strategy for engagement was noted in two studies [20, 35]. Novel programs and interventions can be built from the analyses of qualitative data, which is rich with nuanced information that is not easily expressed as numbers; this includes feelings and preferences about individual learning and gaining knowledge, and idiosyncratic experiences of self-managing diabetes [46]. To ensure comprehensiveness, the voices of older persons—as obtained qualitatively—should not be overlooked in the design, implementation, or evaluation of DSME programs.

#### Gap 4. Limited description of the sustainability of programs

The reported length of the DSME programs was 6 weeks [18, 25], 2 months [26–28], 10 weeks [63], 3 months [10, 12, 15, 30, 36, 59, 60], 5 months [56], 6 months [13, 14, 16, 34, 37, 38, 62], 12 months [57, 64], 18 months [52], and in one program, the duration was determined by patient needs and ranged from 4 months to 2 years [35]. In the reviewed programs/interventions, there was no measure of outcomes maintenance after participation ended. One program was described as ongoing, as the program moved from hospital-based to primary care [32]. Markle Reid et al. [14] recommended considering the sustainability of DSME interventions, instead of only the short-term effects of interventions, which is most often studied.

The sustainability of interventions requires their persistent implementation across settings and delivery sectors, and including attention to health behaviours and outcomes [67]. Maintaining effective programs and practices, especially for older persons with diabetes, is critical for achieving health benefits and policy changes. A lack of a sustainable plan for any intervention jeopardizes future community support, engagement, and trust in researchers [68]. Research is needed to evaluate the effectiveness of these interventions on long-term diabetes-related outcomes. It would be prudent for future DSME programs/interventions to integrate sustainability as a core element for evaluation and research, including the continuation of the DSME components, capacity building, and continued health benefits or outcomes for older persons with diabetes.

#### Gap 5. Scarce description of available education for providers

In only four studies, education was made available to providers in the form of in-service education [4] and formal training for professionals [34] and paraprofessional educators [25, 36]. For example, in Andrich and Foronda’s [4] study, providers received education on DSME and the ADA Standards of Medical Care in Diabetes algorithm to facilitate the project and continue the implementation of DSME after the initial data collection period. The education included evidence-based recommendations, project goals, project plans, written material (i.e., algorithm of care and brochures), and cost analysis with reimbursement opportunities. Based on the review of 40 patient charts, DSME was implemented at a rate of 20% prior to the intervention. Compliance with using DSME information only increased to 35% after the DSME practice change initiative. Although statistically significant (p < 0.05), the results did not meet the objective of the study to increase provider use to 50%. Kellow et al. [34] trained all clinicians in culturally appropriate education approaches that were effective for Chinese patients, including re-orienting their professional positioning in the Chinese-specific therapeutic relationship. In another study, CHWs received 2 months of diabetes education based on the Association of Diabetes Care & Education Specialists’ (ADCES7) Self-Care Behaviors curriculum. CHWs were trained in monitoring vital signs, checking HbA1C and glucose levels at home, documentation, and using the telemedicine videoconferencing platform [36]. In Pauley et al.’s [25] study, personal support workers (PSWs) were trained in diabetes management, including health behaviour change, adherence to treatment, avoidance, reduction of unhealthy behaviours, and adoption of healthy behaviours.

The need for quality diabetes education is well known [10, 25, 36]. HCPs’ misconceptions about diabetes management may have a negative effect on the quality of services provided to older persons with diabetes [10, 34]. In six studies, authors recommended researching the process of education itself [23, 60], the type of education received by HCPs [23, 33], HCPs’ knowledge [10], evaluating the current contents and effectiveness of different delivery modes of DSME [23, 58], and evaluating locally-developed programs and comparing their content to programs developed for studies and guidelines [64].

## Discussion

DSME is a vital component of diabetes care to prevent or delay complications [9, 10, 20, 22, 36, 54]; however, its implementation or the range of programs/interventions in persons aged 65 years and older has not been well documented [46]. This scoping review was conducted to address this gap in knowledge and to map the available evidence about DSME for persons aged 65 years and older living with diabetes in Western countries so that the circumstances and unique needs of this group do not go unnoticed or minimized. This is the first scoping review to focus on describing the aims, type of program, theoretical/philosophical underpinnings, topics covered, program length, outcomes and evidence gaps for persons 65 years and older in Western countries.

DSME, recognized as a crucial element of person-centred care, has been studied by researchers [9, 10, 20, 24, 36, 54]. It aims to empower persons who live with diabetes by equipping them with knowledge, self-care practices, coping skills and shaping attitudes to modify their behaviour and effectively prevent or delay complications [9, 12, 17, 23, 26, 30, 37, 53, 56, 57]. To ensure effectiveness and applicability to older adults, *DSME must account for individuals’ lived experiences*—including the influence of the SDH—while adhering to evidence-based standards of care [17, 20, 23, 24, 29, 36, 37]. By incorporating these factors into the development of DSME programs, health care providers can align education and support with the unique circumstance of older persons, thereby enhancing the overall effectiveness of the intervention or program.

The scoping review revealed a *diverse range of DSME programs or interventions encompassing various designs* [9, 10, 12, 15–18, 26, 30–34, 37, 38, 52, 54–57, 59–64], *theoretical foundations* [9, 10, 12–15, 26, 28, 30, 33, 37, 52, 60, 63], and *education topics* [9, 10, 12, 15, 16, 18, 22, 26, 27, 30–34, 36, 37, 56, 57, 60, 63]. These findings highlight the importance of tailoring interventions or programs to address the specific needs of older persons with diabetes yet seems to suggest an (adaptable) gold standard remains elusive. Technological and non-technological education demonstrated promise for older persons with diabetes. Technologically-driven education included the use of iPads^®^ [33], Fitbit^®^ devices [18], telehealth [36], synchronous sessions [12], and the use of online doctors’ notes [55]. Despite the popularity of these technologies, consideration must be given to older persons of low socioeconomic status who do not have access to smartphones and who may have lower technology literacy or those who live in rural areas where connectivity to the network is often challenging. Ideal interventions must be inclusive and reach populations living in vulnerable conditions. For example, in Marsh et al.’s [36] study, participants accessed telehealth via CHWs visiting their homes, a strategy that guaranteed regular consultation with HCPs and improved clinical, behavioural, and learning outcomes in older persons with lower SDH [36].

Another relevant finding from this review was *education is organized as individual*, *group-based*, *individual and group-based*, *and peer-oriented programs*. Individual education was provided in eight studies [12, 22, 25, 28, 29, 33, 36, 57], peer-mentoring sessions in two studies [27, 35], and group-based programs in 20 studies [9, 10, 15–18, 30–32, 34, 37, 38, 54, 56, 59, 61–64], and individual and group sessions in two studies [13, 14]. In one study, researchers indicated education was initially planned for small group sessions; however, due to low attendance, case-by-case education was instead provided [52]. DSME should be based on the diversity, culture, health literacy levels, levels of independence, comorbidities, and unique needs of the older population. By considering factors, such as delivery mode, cultural relevance, and personalized support, these programs can effectively aid older persons to actively engage in self-management behaviours, leading to improved overall well-being, diabetes management and QoL.

Researchers found DSME programs/interventions to be an effective means of improving *clinical* outcomes (e.g., HbA1C, blood glucose, lipid and renal profile, and BMI), *SMBs* (e.g., dietary habits, physical activity, and coping strategies), and *learning* outcomes (e.g., diabetes knowledge, and self-efficacy) in Western countries. Moreover, the general success of these interventions does not appear to be restricted to specific geographical factors, such as languages, cultures, or how health care systems are organized.

Specific to *clinical* outcomes, many reviewed studies showed significant improvements, particularly HbA1C, in older persons with diabetes who participated in DSME programs [12, 15, 26, 35–38, 56, 57, 59, 60, 63, 64]; notably however, a few studies did not find significant reductions in HbA1C [32, 34]. Other clinical outcomes, such as fasting blood glucose, weight/BMI, cognitive function, blood pressure, lipid profile, waist circumference, waist-to-hip ratio, and renal profile, also improved in some studies [10, 31, 32, 35, 60, 64]. These findings highlighted the potential of DSME programs to enhance overall health and well-being in older individuals with diabetes. Understanding the impact of DSME programs on clinical outcomes and individual preferences contributes to ongoing efforts to optimize diabetes care. Future research should explore the long-term effects and cost-effectiveness of DSME programs, as well as address the preferences and needs of individuals in different health care settings.

The studies also emphasized the positive influence of DSME programs on diabetes *knowledge and self-efficacy*, enhancing older individuals’ understanding of the disease. However, it is important to acknowledge providing education does not guarantee increased knowledge. Self-efficacy outcomes varied, with some studies showing improvements [25, 65] and others showing no significant change [14]. To effectively empower individuals in managing their condition, DSME programs should address both knowledge and self-efficacy. By combining educational interventions with strategies to enhance self-efficacy, DSME programs can promote greater confidence and self-management skills among individuals with diabetes.

DSME programs yielded positive effects on *behavioural* outcomes, fostering improvements in self-management behaviours like dietary habits, physical activity, health care utilization, blood glucose monitoring, medication adherence, and problem-solving skills [11, 12, 14–16, 29, 30, 34–38, 53, 56–59, 65, 66]. These programs enabled individuals, promoting favorable lifestyle changes and equipping them with the necessary tools for effective diabetes management. Moreover, DSME programs enhanced the QoL for older persons with diabetes [10, 14, 16, 62, 65], except for one study which had a trend of declining mental health-related QoL [64]; this underscores the need for tailored interventions to address these concerns. Overall, these findings accentuated the significance of integrating behavioural interventions into diabetes care to bolster clinical outcomes, foster self-management behaviours, improve mental health, and enhance the QoL for older individuals with diabetes. The incorporation of sustainable strategies, continuous research, and customization to individual needs remain imperative for optimizing the effectiveness of DSME programs.

Another key finding of this review was *the lack of measurement of outcomes beyond the program duration*. Ensuring the sustainability of interventions is crucial for achieving long-term health benefits and driving policy changes, especially for older persons with diabetes. Hence, it is vital to assess the longevity (as designed) of effective programs across diverse settings and delivery sectors, while also addressing health behaviours and outcomes. Failing to establish a sustainable plan for interventions can undermine future community support, engagement, and trust in researchers. Therefore, future DSME programs should prioritize sustainability as a core element, integrating it into evaluation and research. This encompasses the continuity of DSME components, capacity building, and the assessment of long-term health benefits and outcomes for older persons with diabetes. Determining the effectiveness of interventions on long-term diabetes-related outcomes is essential to ensure the appropriateness and enduring impact of DSME programs.

The *limited inclusion of educational interventions for HCPs* within the reviewed studies exposed the need for improvement in this area. Enhancing provider education is crucial to address misconceptions and knowledge gaps, equipping them with the necessary tools to effectively aid older individuals with diabetes. By prioritizing comprehensive education for HCPs, health care systems can improve the quality of care and outcomes for this population. Further research is necessary to explore different approaches to provider education, to evaluate the effectiveness of DSME delivery modes, and to assess locally-developed programs. Emphasizing provider education can translate to better delivery of DSME, ultimately improving management and well-being for older persons with diabetes.

The *limited use of qualitative research designs in studying DSME for older individuals* suggests a need for a deeper understanding of their experiences and needs. Qualitative research is critical in developing person-centered DSME programs that address the unique challenges faced by older individuals with diabetes. Findings from qualitative studies indicated, while older persons may find diabetes education beneficial during diagnosis [20, 52], they perceive it as less effective and repetitive in later stages, and some expressed a reduced need for its continuance. Gaining greater insight into why some feel diabetes education (including ongoing instructional support) may not be applicable or needed, coupled with strategies to ensure continuous *relevant* DSME for older persons, are crucial to maximize and sustain self-management capabilities and prevent complications and disease progression. The incorporation of qualitative data enables researchers to gain valuable insights into the preferences and experiences of older individuals, facilitating the development of tailored interventions that align with their learning styles, literacy levels, culture, and individual circumstances. By integrating the perspectives of older individuals in the design, implementation, and evaluation of DSME programs, more meaningful and relevant interventions can be created, ultimately leading to improved self-management behaviours and outcomes for this population.

Finally, given that only in one study [20] authors described participants’ understanding of DSME, future research should prioritize investigating how older persons define and understand DSME, with appreciation that one can have difficulty fully detailing something to which they are not aware. Importantly, it is about how older persons may have different perspectives and priorities when it comes to managing their diabetes and understanding their unique experiences and perspectives can inform the development of more effective interventions. Exploring the ways in which older persons define and understand DSME can also highlight potential gaps or misconceptions that may need to be addressed in educational programs. As such, by including older persons’ voices, research can contribute to improving the quality and accessibility of DSME interventions and better support older persons in managing their diabetes and improving health outcomes and quality of life.

### Study strengths and limitations

This study has several strengths, including the following. First, to our knowledge, it is the only scoping review that has explored this topic in this context. As such, by describing DSME aims, type of programs, theoretical/philosophical underpinnings, topics covered, program lengths, and outcomes, we have mapped existing evidence in regard to persons 65 years and older living with diabetes in Western countries. This important new contribution aids educators, practitioners, and researchers alike in more effectively accessing the evidence. Further, it has made visible where knowledge gaps exist for this population, providing cautionary note regarding current information, as well as research direction going forward. Second, it has been conducted in a rigorous manner, using a recognized, credible methodological approach. Third, the information has been presented such that there is complete transparency and substantive detail about the process and resulting findings, which also contributes to rigor, replicability, and evidence access.

The limitations of this review are also important to acknowledge. Due to time and resource constraints, we included only articles published in English journals from 2000 to 2022, thereby excluding useful information that may be available in other languages. It is plausible some studies were missed based on the search terms and databases. A general limitation of scoping reviews is the inclusion of articles irrespective of their quality [69]; notably, while the quality of our included studies was not evaluated, this approach was the most appropriate given our initial goal of mapping the literature to determine the current status of research about DSME and older adults before proceeding. Overall, despite these limitations, we believe the review provides an important overview of this evidence, which is an essential phase in advancing practice, policy, and future research. We consider this information of value as it can raise awareness and inform the development and implementation of DSME for older adults in Western countries.

## Conclusion

This scoping review aimed to answer the following question: what has been reported in the literature about DSME for persons aged 65 years and older living with T1D and T2D? It is clear diverse educational approaches have been used over time to engage older persons in DSME. A consistent concern about most of the studies was the lack of representation and engagement of older persons in the definition, development, implementation, and evaluation of DSME. To be inclusive—and as such, to be ‘right’—diabetes education must account for the uniqueness of each person and their circumstance, culture, preferences, experiences, and knowledge; as well, it must accommodate different degrees of independence and comorbidity. With attention to these factors, more substantive and sustained results will be achievable.

## Supporting information

S1 Appendix PRISMA-ScR checklist. (DOCX)

S2 Appendix Sample search strategy. (DOCX)
