# Supplementary material for: Diabetes self-management education (DSME) for older persons in Western countries: A scoping review
Source: PLoS One. 2023 Aug 9;18(8):e0288797. doi: 10.1371/journal.pone.0288797 (PMC10411808; doi:10.1371/journal.pone.0288797)
Supplement: S2 Appendix — (DOCX) [file pone.0288797.s002.docx]

**Supplement 2: Search strategies**

**Table 1: Search strategy MEDLINE**

| **Search** | **Query** |
| --- | --- |
| S1 | diabetes mellitus or diabetes or diabetes type 1 or diabetes type 2 or t2d or t1d or t2dm or t1dm or metabolic syndrome).mp. [mp=title, book title, abstract, original title, name of substance word, subject heading word, floating sub-heading word, keyword heading word, organism supplementary concept word, protocol supplementary concept word, rare disease supplementary concept word, unique identifier, synonyms, population supplementary concept word, anatomy supplementary concept word] |
| S2 | (diabetes education or diabetes training or diabetes knowledge or health education or health literacy or health promotion or diabetes training).mp. [mp=title, book title, abstract, original title, name of substance word, subject heading word, floating sub-heading word, keyword heading word, organism supplementary concept word, protocol supplementary concept word, rare disease supplementary concept word, unique identifier, synonyms, population supplementary concept word, anatomy supplementary concept word] |
| S3 | older adults or elderly or geriatric or geriatrics or aging or senior or seniors or older people or aged 65 or 65+).mp. [mp=title, book title, abstract, original title, name of substance word, subject heading word, floating sub-heading word, keyword heading word, organism supplementary concept word, protocol supplementary concept word, rare disease supplementary concept word, unique identifier, synonyms, population supplementary concept word, anatomy supplementary concept word] |
| S4 | (self-management or self-care or self-regulation or self-monitoring).mp. [mp=title, book title, abstract, original title, name of substance word, subject heading word, floating sub-heading word, keyword heading word, organism supplementary concept word, protocol supplementary concept word, rare disease supplementary concept word, unique identifier, synonyms, population supplementary concept word, anatomy supplementary concept word] |
| S5 | S1 AND S2 AND S3 AND S4 AND S5 |
| S6 | Limit S5 to (English language and yr=”2000 – 2022”) |
